# GraphCpG: imputation of single-cell methylomes based on locus-aware neighboring subgraphs

**DOI:** 10.1093/bioinformatics/btad533

**Published:** 2023-08-30

**Authors:** Yuzhong Deng, Jianxiong Tang, Jiyang Zhang, Jianxiao Zou, Que Zhu, Shicai Fan

**Affiliations:** School of Automation Engineering, University of Electronic Science and Technology of China, Chengdu 611731, Sichuan, China; School of Automation Engineering, University of Electronic Science and Technology of China, Chengdu 611731, Sichuan, China; School of Automation Engineering, University of Electronic Science and Technology of China, Chengdu 611731, Sichuan, China; School of Automation Engineering, University of Electronic Science and Technology of China, Chengdu 611731, Sichuan, China; Shenzhen Institute for Advanced Study, University of Electronic Science and Technology of China, Shenzhen 518110, Guangdong, China; Department of Out-patient, The Second Affiliated Hospital of Chongqing Medical University, Chongqing 400010, China; School of Automation Engineering, University of Electronic Science and Technology of China, Chengdu 611731, Sichuan, China; Shenzhen Institute for Advanced Study, University of Electronic Science and Technology of China, Shenzhen 518110, Guangdong, China

## Abstract

**Motivation:**

Single-cell DNA methylation sequencing can assay DNA methylation at single-cell resolution. However, incomplete coverage compromises related downstream analyses, outlining the importance of imputation techniques. With a rising number of cell samples in recent large datasets, scalable and efficient imputation models are critical to addressing the sparsity for genome-wide analyses.

**Results:**

We proposed a novel graph-based deep learning approach to impute methylation matrices based on locus-aware neighboring subgraphs with locus-aware encoding orienting on one cell type. Merely using the CpGs methylation matrix, the obtained GraphCpG outperforms previous methods on datasets containing more than hundreds of cells and achieves competitive performance on smaller datasets, with subgraphs of predicted sites visualized by retrievable bipartite graphs. Besides better imputation performance with increasing cell number, it significantly reduces computation time and demonstrates improvement in downstream analysis.

**Availability and implementation:**

The source code is freely available at https://github.com/yuzhong-deng/graphcpg.git.

## 1 Introduction

DNA methylation, a covalent modification frequently occurring at cytosine–guanine dinucleotides (CpGs), is the best characterized epigenetic mark associated with biological processes such as aging and tumorigenesis (Horvath 2013, Schübeler 2015, Dor and Cedar 2018, Seale *et al.* 2022).

To measure single-cell DNA methylation levels, several protocols have been proposed for the last decades. Although conversion-free methods continue to develop, the method based on bisulfite conversion is still taken as the gold standard for profiling DNA methylation (Ahn *et al.* 2021, Niemöller *et al.* 2021). Methods of this type include reduced representation bisulfite sequencing (scRRBS-Seq) (Guo *et al.* 2013), the single-cell bisulfite sequencing (scBS-Seq) (Smallwood *et al.* 2014), and single-nucleus methylcytosine sequencing (snmC-seq) (Luo *et al.* 2017). These protocols have made it possible to explore the inter-cellular heterogeneity and detail dynamics of DNA methylation in single cells (Farlik *et al.* 2015, Angermueller *et al.* 2016).

However, the small amount of DNA available per cell compromises the coverage of measurements, which leads to 60%–99% missing values for scBS-seq, scRRBS-seq, and snmC-seq (Angermueller *et al.* 2017, Luo *et al.* 2017, Kapourani and Sanguinetti 2019). High sparsity hinders downstream understanding of underlying biological processes at the level of the whole genome. Thus, imputation techniques are necessary to address the inherent sparsity of single-cell methylation data.

Genome-wide imputation of single-cell methylation status has been established well in the past to impute sparse data by traditional machine-learning models and deep learning models. To impute methylation status in scale of single CpG site, varying types of methods were taken into processing.

Based on traditional machine learning, LightCpG (Jiang *et al.* 2019) combines the CpG positional features with DNA sequence and additional CpG islands structural features to feed a LightGBM model for imputing. Melissa (Kapourani and Sanguinetti 2019), a Bayesian hierarchical method, imputes unassayed CpG sites by leveraging local correlations between neighboring CpGs and similarity among cells. Our in-house method, CaMelia (Tang *et al.* 2021) extracts the locally paired similarity of inter-cellular methylation patterns, DNA sequence feature, and intra-cellular neighboring methylation patterns for the CatBoost gradient boosting model to predict states. It achieved state-of-the-art performances over traditional machine-learning methods.

With deep learning prevalent, based on deep neural networks (DNN), DeepCpG (Angermueller *et al.* 2017) utilizes DNA sequence patterns and methylation states to predict methylation states, namely associations between genome and methylation as well as between neighboring CpG sites. Based on a multi-task architecture, it joins DNA and CpG from a convolutional neural network (CNN) module and a recurrent neural network (RNN) module, respectively. Inspired by Transformers (Vashishth *et al.* 2020), CpG Transformer (De Waele *et al.* 2022) predicts methylation status using a 2D sliding window self-attention, whose inputs include the CpG matrix along with CpG positions and corresponding DNA sequence.

According to the similar operation of distilling the neighboring methylation and genome information, current works reach a consensus on the importance of mining neighbors of the target CpG site. Traditional machine-learning methods like LightCpG significantly shorten the training time, but they left room to improve imputation performance. Though CaMelia achieved better imputation performance, its preprocessing on manufactural locally paired similarity feature takes an impractically long time on large datasets. Conversely, DNN-based methods, such as the latest CpG Transformer, obtained sound results. However, with the increasing number of cells, its quadratically scaled model limits scalability on the coming single-cell methylation studies whose cell numbers could be very large. Additionally, its combining encoding of CpG, cell index, and DNA context bloats the model even larger. With the trend of the increasing volume of single-cell DNA methylation datasets (Tian *et al.* 2022), recent large datasets could even have higher sparsity on their raw data, especially methylation matrices sequenced over the whole genome on a larger number of cells at the same time. To impute methylation state on such sparse and large datasets, a framework for accurate imputation on large datasets is in need.

To address these issues, we proposed GraphCpG, a graph-based deep learning method using locus-aware neighboring subgraphs to impute the missing methylation states. Based on neighboring subgraphs encoded with distinguishable neighboring locus and identical cells, we then utilized a graph-based neural network to generate an optimized representation for the target methylation state, which consolidates follow-up neural networks in prediction. In the test of highly sparse real datasets with a large number of cell samples (Farlik *et al.* 2016, Luo *et al.* 2017), GraphCpG obtained state-of-the-art imputation results. It also got competitive results on denser datasets whose cell number is relatively small (Hou *et al.* 2016, Kretzmer *et al.* 2021). Each locus-aware neighboring subgraph of the predicted target site can be visualized as a bipartite graph, whose nodes are able to be traced back and analyzed for inter-cellular and inter-loci similarity using generated optimized representation. In contrast to previous approaches, GraphCpG only utilizes methylation matrices for learning dependencies among methylation sites in a general data-driven manner. Without CpG position information and DNA context, the completion of the methylation matrix is transformed into a graph-based link prediction problem in a non-Euclidean space and the computational complexity is also reduced. Compared with other available methods on the latest datasets, it costs a shorter time. Furthermore, by accurately imputing missing values in single-cell methylation data, it enhances the performance of cell clustering, cell type identification, and differential methylation analysis.

## 2 Materials and methods

GraphCpG addresses the missing problem by only focusing on mining inter- and intra-cellular neighboring CpGs. Meanwhile, inspired by the inductive local graph pattern (Zhang and Chen 2020) and simplified role-aware feature (Shen *et al.* 2021), locus-aware encoding is proposed to inductively hasten learning of the neighboring subgraph which indicates the missing methylation state.

**Figure 1.**
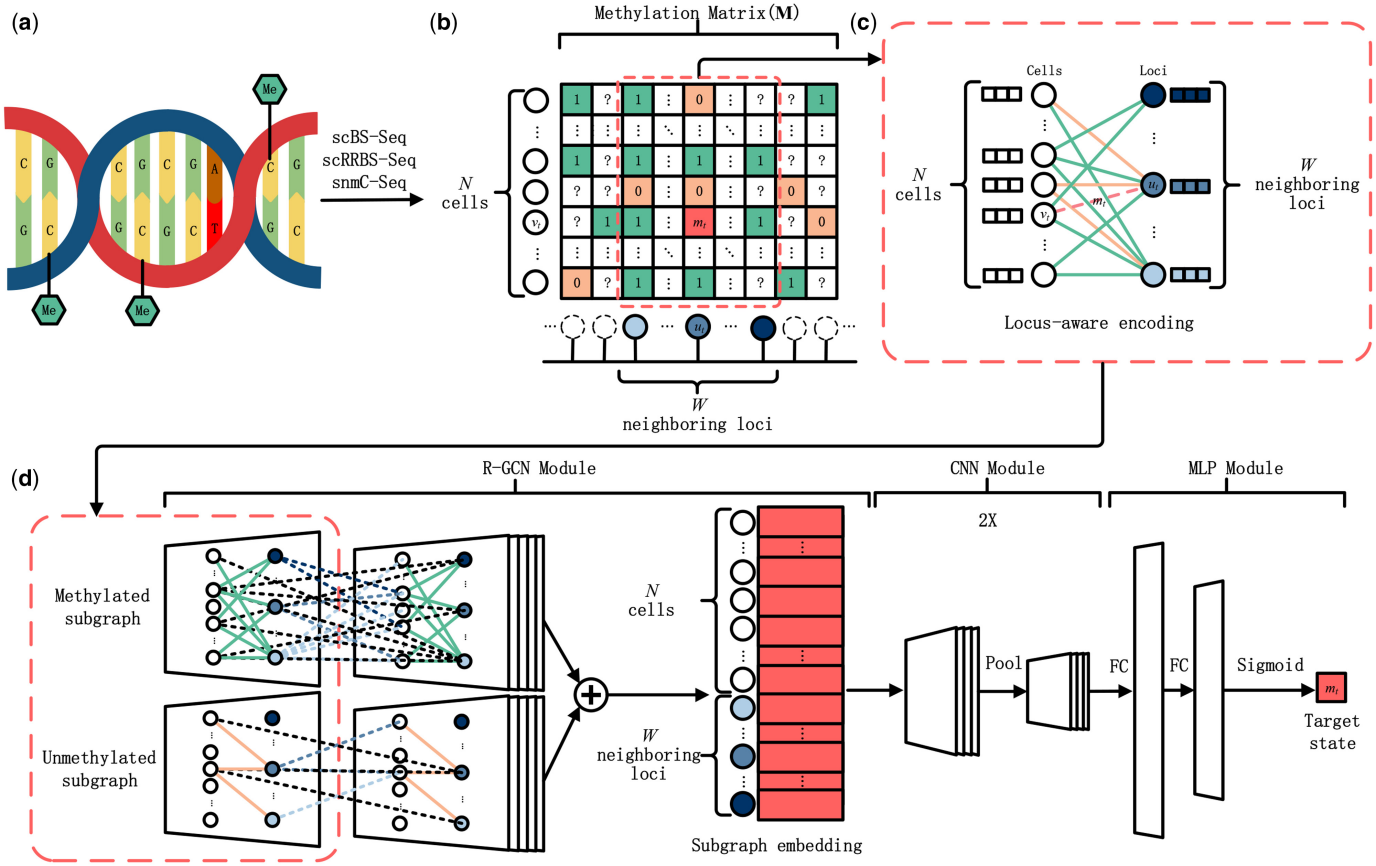
The overview of GraphCpG. (a) Single-cell CpG profiles. The cytosine-5 methylations within the CpG dinucleotides are marked as Me. (b) Neighboring subgraph extraction. A subgraph is extracted by a sliding window around each interesting methylation state, whose cells, loci, and methylation states are applied row-wise, column-wise, and element-wise. (c) Locus-aware encoding. The masked target methylation state is profiled by the locus-aware encoding of the studied general cell type and different loci. (d) Modular architecture of graph neural network. A sequential architecture consists of a relational graph convolution network (R-GCN) module, a convolutional neural network (CNN) module, and a multi-layer perceptron (MLP) module.

As shown in Fig. 1a, the single-cell DNA methylation profiles used in this study are sequenced by scBS-seq, scRRBS-seq, or snmC-seq (Angermueller *et al.* 2017, Luo *et al.* 2017, Kapourani and Sanguinetti 2019). In Fig. 1b, missing, unmethylated, methylated, and target states of CpG sites are denoted by white, orange, green, and red squares, respectively. The neighboring subgraph indicating the target state is selected by a red dashed window sliding along the loci. Then, locus-aware encoding anonymously initializes sequential loci and the role of an abstract cell type for each subgraph. Different encodings are drawn in blue gradient and white individually (Fig. 1c). To learn the bimodal distribution of methylated sites and unmethylated sites on the heterogeneous neighboring subgraph (Rakyan *et al.* 2004, Fan and Chi 2016), the subgraph is separated into methylated subgraph and unmethylated subgraph, one for all methylated sites and one for all unmethylated sites. As the consecutive architecture in Fig. 1d, a relational graph convolution network (R-GCN) (Schlichtkrull *et al.* 2018) module passes encodings to subsequent layers based on methylated subgraph and unmethylated subgraphs, respectively. A combination of CNN and multi-layer perceptron (MLP) then compresses subgraph embedding from the previous graph neural network (GNN) to predict the methylation state at the target site. Specifically, the CNN module consists of two convolutional and pooling layers, and the MLP module predicts based on two fully connected layers and a sigmoid activation function.

### 2.1 Model inputs

The input to GraphCpG is a G denoting the undirected bipartite graph constructed from the methylation matrix M. In G, a node related to an interesting CpG state is either a cell u (a row in M) or a locus v (a column in M). Namely, M is the adjacency matrix of G, which is constructed by methylation states (links): 1 for methylated state and 0 for unmethylated state. Methylation states link cells and locus, which represents the fact whether a locus in a cell is methylated or unmethylated. Each methylation state, as an edge (u,v), has an encoding $m=M_{u,v}$, corresponding to the methylation state on a locus v expressed in a cell u. Encoding set of unmethylated and methylated states is M={0,1} respectively after binarizing observed elements of M. Neighbors of u with edge type m are denoted as $N_{m}(u)$.

### 2.2 Neighboring subgraph extraction

Initially, neighboring subgraphs are extracted in the data processing. As shown in Fig. 1b, a window slides horizontally by locus, whose height is the total number of cells N and width is the empirical neighbor range W centered around the target locus $u_{t}$. By traversing all the observed methylation states from M, the neighboring subgraph is extracted related to each target pair $\left(u_{t},v_{t}\right)$ with $m_{t}$ removed. These neighboring subgraphs are delivered to the locus-aware encoding part later. Similarly, during testing, for each $\left(u_{t},v_{t}\right)$ missing methylation state, the same extracting process is performed before subsequent prediction.

### 2.3 Locus-aware encoding

The second part of GraphCpG is locus-aware encoding (Fig. 1c). During loading subgraphs to the GNN, locus-aware encoding is applied. To identify the different roles of nodes in a subgraph, various integer labels are used to encode u and v, respectively. Starting from all the cells encoded the same, sequential loci are then encoded with consecutive integers to identify different relative distances between W neighboring loci and the target locus. Since the target locus is at the center of the neighboring loci, the W should be an odd number. Notably, all the cells from a dataset are encoded with the same feature, which represents the general cell type of the dataset. By one-hot encoding of these different integers inside each neighboring subgraph, the GNN can differentiate the interactions from locus to cell, the interactions from cell to locus, and the interactions among different loci.

### 2.4 Modular architecture of graph neural network

The training part of GraphCpG is a sequential model predicting methylation states based on the encoded neighboring subgraphs. The architecture of this model consists of an R-GCN module to extract a feature vector for each role in the subgraph, a CNN module to compress the feature, and an MLP module to output the predicted methylation state (Fig. 1d).

In this architecture, GNN’s message-passing layers are implemented by the R-GCN (Schlichtkrull *et al.* 2018), which can learn the rich subgraph patterns introduced by multiple edge types (Zhang and Chen 2020). The rich subgraph patterns include the average methylation state related to loci and cells individually, the total amount of m connected to the target locus or cell, and similar cells’ average methylation states, etc. The message-passing form is given by the following equations:

(1) $$x_{i}^{l+1}=\sigma \left(\sum_{m\in M}\sum_{j\in N_{m}(i)}\frac{1}{\left|N_{m}(i)\right|}W_{m}^{l}x_{j}^{l}+W_{0}^{l}x_{i}^{l}\right)$$

where $x_{i}^{l}\in R^{d^{l}}$ is the feature vector of node i∈{1,…,N+W} at layer l, with $d^{l}$ being the dimensionality of this layer’s representations. $\left\{W_{m}^{l}|m\in M\right\}$ and $W_{0}^{l}$ are learnable parameter matrices. To simplify, the bias term is left out of the notation. Learning the enriched graph patterns is enabled by different parameter matrices $W_{m}^{l}$, which processes $N_{m}(i)$ denoting neighbors j connected to i under different relation m∈M. $\left|N_{m}(i)\right|$ is chosen as the normalization constant for the aggregation among neighbors. During stacking L message-passing layers, messages are accumulated and passed through an element-wise activation function σ(⋅), such as tanh(⋅) here. By simply concatenating node i’s feature vectors from each layer as (Xu *et al.*, 2019), the final representation of the node i is obtained:

(2) $$\begin{array}{c} h_{i}=[x_{i}^{1},x_{i}^{2},\ldots x_{i}^{L-1},x_{i}^{L}]\\ D=\sum_{l\in L}d^{l} \end{array}$$

D is the total length of the node i’s feature vector. The optimized representation $H_{u_{t},v_{t}}\in R^{(N+W)\times D}$ related to a missing CpG site $\left(u_{t},v_{t}\right)$ is concatenated by node i’s representations from a neighboring subgraph which has N cells and W locus:

(3) $$H_{u_{t},v_{t}}=\left[h_{1},h_{2},\ldots h_{i},\ldots h_{N+W-1},h_{N+W}\right]$$

Next, we generate the graph-level vector by a CNN module. The input representation $H_{u_{t},v_{t}}$ is transformed by a 2D-convolutional layer with the kernel representation $k_{i,d}$ related to each position (i,d), which computes the compressed representation followed by an activation function ReLU(⋅) and a max pooling layer Maxpool(⋅):

(4) $$g=\text{Maxpool}(\text{ReLU}(\sum_{i=1}^{N+W}\sum_{d=1}^{D}w_{\mathrm{fid}}k_{i,d}))$$

where $w_{\mathrm{fid}}$ are the parameters of the convolutional filter f for the kernel $k_{i,d}$. After reshaping the compressed graph representation, an MLP and a sigmoid activate function are used to output the predicted methylation state:

(5) $${\hat{m}}_{u_{t},v_{t}}=\text{sigmoid}(w^{⊤}\text{ReLU}(gW^{⊤}))$$

A scalar methylation state ${\hat{m}}_{u_{t},v_{t}}\in (0,1)$ is obtained by the MLP, which has one fully connected hidden layer with parameters W, a ReLU activation function, and the other fully connected hidden layer with parameters w.

### 2.5 Model training

Model parameters were learned on the training set by minimizing the binary cross entropy (BCE) loss function between the predictions and the ground truth methylation encodings:

(6) $$L=-\frac{1}{\left\{(u_{t},v_{t})|\Omega_{u_{t},v_{t}}=1\right\}}\sum_{(u_{t},v_{t}):\Omega_{u_{t},v_{t}}=1}\;\left[m_{u_{t},v_{t}}\;\text{log}\;\left({\hat{m}}_{u_{t},v_{t}}\right)+\left(1-m_{u_{t},v_{t}}\right)\;\text{log}\;\left(1-{\hat{m}}_{u_{t},v_{t}}\right)\right]$$

where $\left(u_{t},v_{t}\right)$ indicates the observed methylation states of the methylation matrix M masked by 0/1 mask matrix $\Omega_{u_{t},v_{t}}$. Additionally, $m_{u,v}$ and ${\hat{m}}_{u,v}$ denote the ground truth methylation state and predicted methylation state of target $\left(u_{t},v_{t}\right)$ individually.

### 2.6 Datasets

As shown in Table 1, five public datasets were applied in this study, which came from four single-cell methylation experiments with the number of cells increased (Farlik *et al.* 2016, Hou *et al.* 2016, Luo *et al.* 2017, Kretzmer *et al.* 2021). The first three datasets are the same as those used in CpG Transformer and the rest two are processed from raw profiles with similar pipelines. Compared with the first two datasets based on RRBS, the last three whole-genome datasets have higher sparsity. All datasets are detailed at the level of chromosomes in Supplementary Table S1.

**Table 1.** Summary of datasets statistics.

Datasets	Cell number	Sum sites	Observed sites	Sparsity (% unobserved)^a^
HCC	25	2 044 635	5 891 193	88.47
MBL	30	4 779 569	13 155 172	90.83
Hemato	122	18 050 756	34 855 325	98.42
Neuron-Mouse	690	19 974 995	414 017 690	97.00
Neuron-Homo	780	26 977 898	746 556 608	96.45

a The sparsity is the proportion of unobserved sites on all the sites.

The first dataset (GSE65364) consists of 25 human hepatocellular carcinoma cells (HCC) profiled using scRRBS-seq (Hou *et al.* 2016). The second dataset (GSE125499; sc05) is made of 30 human monoclonal B-cell lymphocytes (MBL) profiled by scRRBS-seq (Kretzmer *et al.* 2021). The third dataset (GSE87197) comprises 122 hematopoietic stem cells (Hemato) and progenitor cells using scBS-seq (Farlik *et al.* 2016). The fourth and fifth datasets (GSE97179; AD008), profiled by snmC-seq, contain 690 mouse neurons (Neuron-Mouse) and 780 human neurons (Neuron-Homo) (Luo *et al.* 2017). These datasets are represented in short as HCC, MBL, Hemato, Neuron-Mouse, and Neuron-Homo. The corresponding genome builds of these datasets are GRCh37 (hg19), GRCh37 (hg19), GRCh38 (hg38), GRCm38 (mm10), and GRCh38 (hg38) respectively.

### 2.7 Experiments

We utilized the same validation method as DeepCpG (Angermueller *et al.* 2017). In our experiment, the CpG sites in the validation set were from chromosomes 13, 14, 15, 16, 17, 18, and 19, and those in the test set were from chromosomes 2, 4, 6, 8, 10, and 12. The CpG sites from the rest chromosomes were used as the training set. Additionally, we binarized the methylation states by rounding off the ratio of methylated read counts to total read counts. For each dataset, GraphCpG only utilized the CpGs methylation matrix as the input, while DeepCpG (Angermueller *et al.* 2017), CaMelia (Tang *et al.* 2021), and CpG Transformer (De Waele *et al.* 2022) additionally used the CpGs positional sequence and the DNA sequence for achieving their best imputation performance.

In our architecture, we adopted an R-GCN with six layers having 32, 64, 128, 128, 64, 32 hidden dimensions individually. The number of parameters $W_{m}$ was reduced by a two-bases basis decomposition (Schlichtkrull *et al.* 2018). Twenty percent of adjacency matrix entries were dropped out randomly for each neighboring subgraph (Zhang and Chen 2020). The loss function was optimized by an Adam optimizer with linear warmup and iterative decay of the learning rate. During training epochs, the model parameter with the lowest validation loss was set for the final model.

As for the first three datasets, training hyperparameters for all the models were the same as the reproduce by De Waele *et al.* (2022). To fully squeeze the performance on one 3090Ti GPU, slight adjustments were made for the following two large datasets, Neuron-Mouse and Neuron-Homo. Because the sliding window width W and the batch size were also coupled in influencing memory consumption on the GPU, W and the batch size were tailor-made individually for each dataset. Normally, 21 neighboring loci are practicable for these datasets and a grid search of this parameter is detailed in Supplementary Table S2. Specific implementations for all the models are detailed in Supplementary Table S3.

## 3 Results

### 3.1 Performance comparison

To evaluate the performance of our GraphCpG, comparisons were performed among two deep learning methods, DeepCpG and CpG Transformer, and our previous traditional machine-learning method, CaMelia.

In Table 2, GraphCpG, though only utilized methylation matrices in training, achieved 96.99 and 89.73 on HCC and MBL for all the cells, which are competitive values of the area under the receiver operating characteristic curve (AUROC). It surpassed other models on Hemato, Neuron-Mouse, and Neuron-Homo datasets with AUROC at 89.77, 91.75, and 93.2, respectively (Supplementary Fig. S1). Corresponding results are also observed on Matthews correlation coefficient (MCC) score, macroF1, and Balanced accuracy (Supplementary Table S4). Besides, similar advances were obtained in contexts such as regulatory elements and CpG islands, which are associated with facilitating the further discovery of cell heterogeneity (Supplementary Fig. S2).

**Table 2.** The performance comparison of GraphCpG with other methods on different datasets.^a^

Dataset	Cell number	AUROC				MCC score			
			Deep learning				Deep learning		
		CaMelia	DeepCpG	CpG Transformer	GraphCpG	CaMelia	DeepCpG	CpG Transformer	GraphCpG
HCC	25	97.11	96.01	**97.56**	96.99	83.32	78.71	**84.43**	81.61
MBL	30	89.36	87.12	**92.05**	89.73	63.17	60.09	**70.58**	64.71
Hemato	122	87.68	88.26	89.56	**89.77**	69.04	67.96	68.15	**69.05**
Neuron-Mouse	690	91.13	88.59	90.87	**91.75**	71.05	66.52	70.77	**71.1**
Neuron-Homo	780	92.98	90.06	92.31	**93.2**	75.01	73.85	75.15	**75.24**

a Bold numbers indicate the best performance.

**Figure 2.**
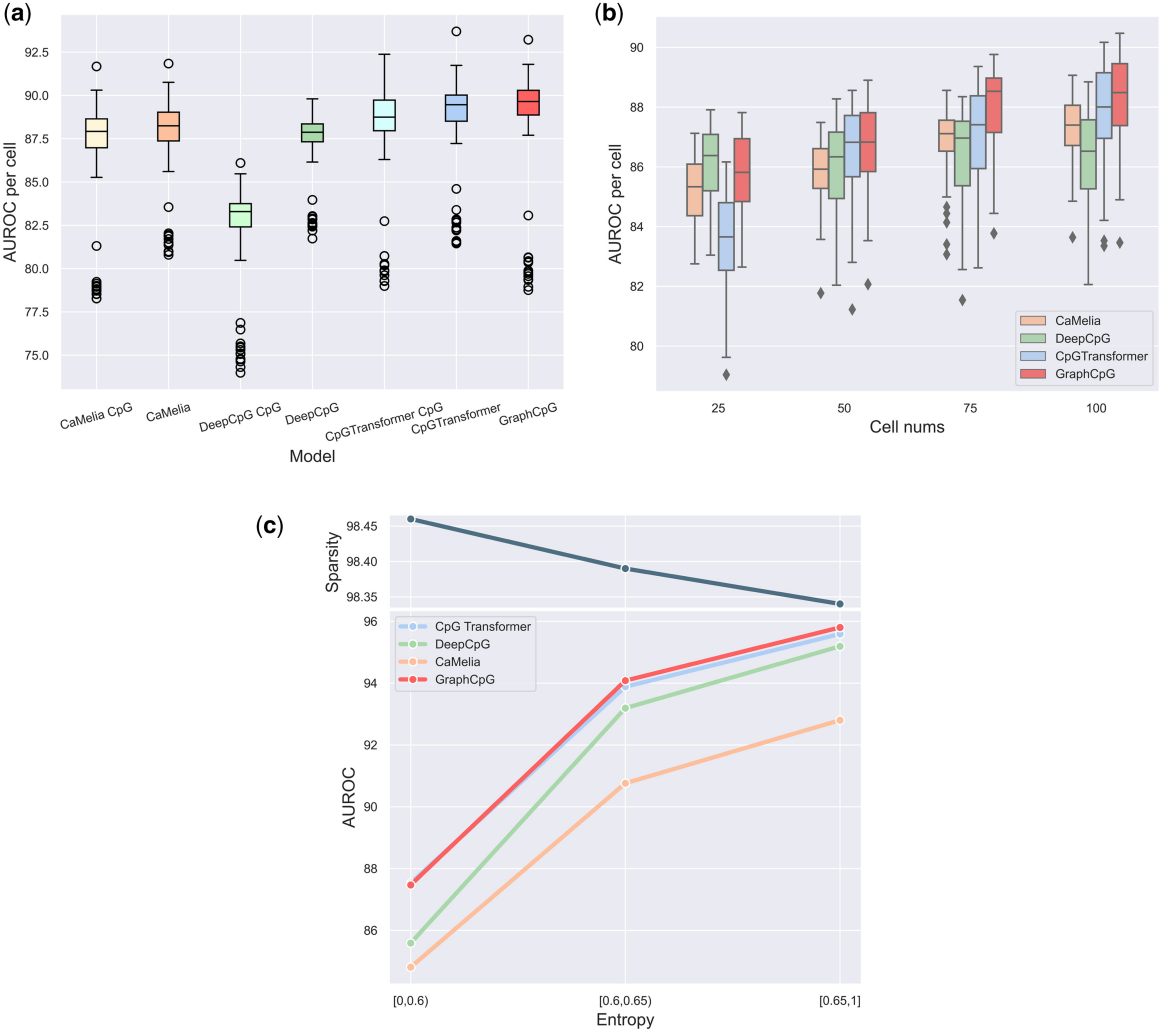
Performance comparison. (a) Comparison of performance by cell on Hemato including compared models trained using only CpG matrices (CaMelia CpG, DeepCpG CpG, CpGTransformer CpG). (b) Performance of models per cell on the datasets sampled by different numbers of cells from Hemato. (c) Performance of models in the function of entropy of windows. The top line chart indicates average sparsity changing with different entropy.

To compare fairly and assess the influence of DNA sequence and CpGs positional sequence, we trained the other models merely using neighboring methylation matrices (CaMelia CpG, DeepCpG CpG, CpGTransformer CpG). In Fig. 2a, GraphCpG demonstrates more advances when all the models exclusively use CpG methylation matrices. As for compared models, though DNA sequence and CpGs positional sequence enhance their prediction, GraphCpG still outperforms them trained with both CpG and DNA features.

To explore how an increasing number of cells improve methods respectively, we trained all the models on four individual datasets by randomly subsampling 25, 50, 75, and 100 cells from Hemato. In Fig. 2b, with the number of cells increasing, the performance of all the machine-learning models is improved. This improvement indicates more cell samples can promote the imputation performance of unknown CpG sites and ameliorate the sparsity problem. Furthermore, the performance of GraphCpG rises and outperformed others on datasets having more cells. The advances are probably due to it strengthening the extraction of local similarity between cells using deep learning based on subgraphs rather than manual feature engineering as in CaMelia.

Massive samples can help researchers restore methylation matrices. However, methylated and unmethylated sites could be highly mixed up in some sliding windows in this case, which is hard for models to predict. To evaluate the performance of models on these highly mixed-up windows, we performed predictions on windows by different entropy on Hemato. Meanwhile, we recorded sparsities as in the upper part of Fig. 2c. The results indicate windows become denser with higher entropy. Below, GraphCpG surpassed all other models after the entropy of windows larger than 0.6. A potential explanation for the advance is message passing along subgraph links, which enables reliable information to pass among nodes of neighboring loci and cells in chaos.

### 3.2 Study of GraphCpG

We conducted an ablation study based on all the datasets to study the locus-aware encoding in GraphCpG, as listed in Table 3. To be specific, we are interested in different levels of component awareness in the neighboring subgraph, which indicate the importance of locus-aware encoding. The original model can identify different loci and cells depicting one general cell type. Without locus-aware encoding, the model can only identify the cells and the loci without being aware of each specific locus. Obvious descents on all the datasets demonstrate the importance of distinguishing different loci by relative position in the matrix. Without any encodings, the model erases all the identity information by setting all the nodes representing loci or cells with the same encoding. Compared with previous encoding ways, the nuance demonstrates the slight contribution of merely distinguishing the general cell type from the locus.

**Table 3.** Ablation study on all the datasets (AUROC).^a^

Model	GraphCpG	Without locus-aware encoding	Without any encoding
HCC	**96.99**	95.87	95.87
MBL	**89.73**	86.38	86.21
Hemato	**89.77**	87.33	87.31
Neuron-Mouse	**91.75**	90.15	90.25
Neuron-Homo	**93.2**	91.92	91.89

a The original model is compared with models simplifying encoding type step by step. Bold numbers indicate the best performance.

**Figure 3.**
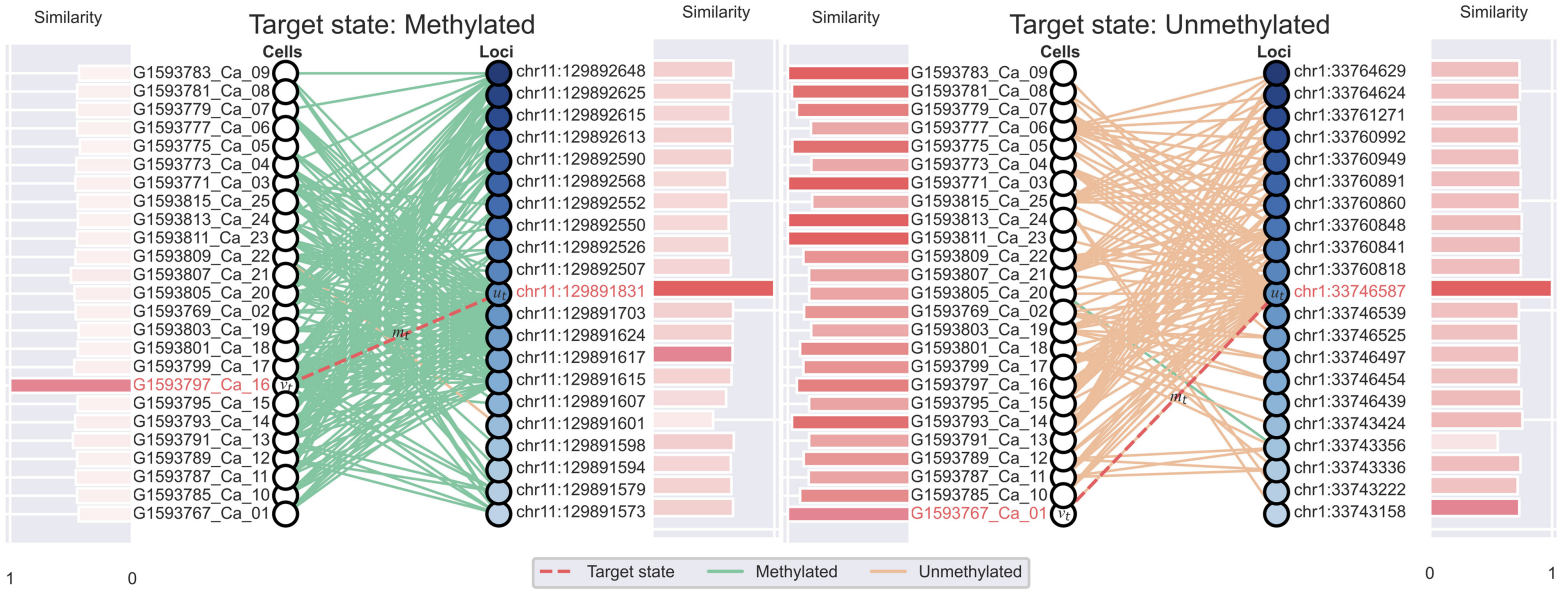
Visualization of locus-aware neighboring subgraphs from HCC. The left graph represents a methylated target site and the right graph profiles an unmethylated target site. For each bipartite graph, the left sides are cells dyed in white with the same encoding, and the right sides are sequential loci dyed in a blue gradient with sequential encoding. Cell labels and locus positions are retrieved and labeled on side of each node. On the left sides of cell nodes are similarities between the subgraph embedding of the target cell and the subgraph embeddings of other cells. On the right sides of loci nodes are similarities between the subgraph embedding of the target locus and subgraph embeddings of neighboring loci.

Equipped with locus-aware encoding, neighboring subgraphs lend themselves well to be visualized by bipartite graphs as in Fig. 1c. Additionally, the original cell and locus position can be retrieved. Furthermore, based on the subgraph embedding obtained by GNN as in Fig. 1d, cosine similarities were calculated both between the target cell and other cells, as well as between the target locus and neighboring loci. We combined the above components to compare the difference between methylated neighboring subgraphs and unmethylated neighboring subgraphs. As visualized in Fig. 3, the top methylated and unmethylated neighboring subgraphs from HCC are illustrated in comparison, which are ranked by model outputs.

Compared with the neighboring subgraphs of unmethylated sites, the neighboring subgraphs of methylated sites have more methylated sites (links) and dramatically distinct patterns. Methylated patterns typically have high cell average methylation and high locus average methylation. In contrast, unmethylated patterns have low locus average methylation and low cell average methylation. Besides, on datasets such as Hemato, the unmethylated pattern would have mixed methylation states on loci in cells as mentioned in Section 3.1 (Supplementary Fig. S3). A likely explanation for this chaos is the large number of cell samples, which makes each locus corresponding to more different states in cells. As for similarities among node embeddings of both cells and loci, nodes having similar values would have similar link patterns to the opposite partition, and vice versa (e.g. chr1:33746587 and chr1:33743356 in the right bipartite graph of Fig. 3). Enabled by these embeddings, GraphCpG filters out cells and loci that are similar to each other, which allows for a more accurate prediction of the target state between the target locus and the target cell.

### 3.3 Computation boost

We evaluated the computation boost of GraphCpG on datasets with increasing numbers of cells and proportions of used loci, individually.

**Figure 4.**
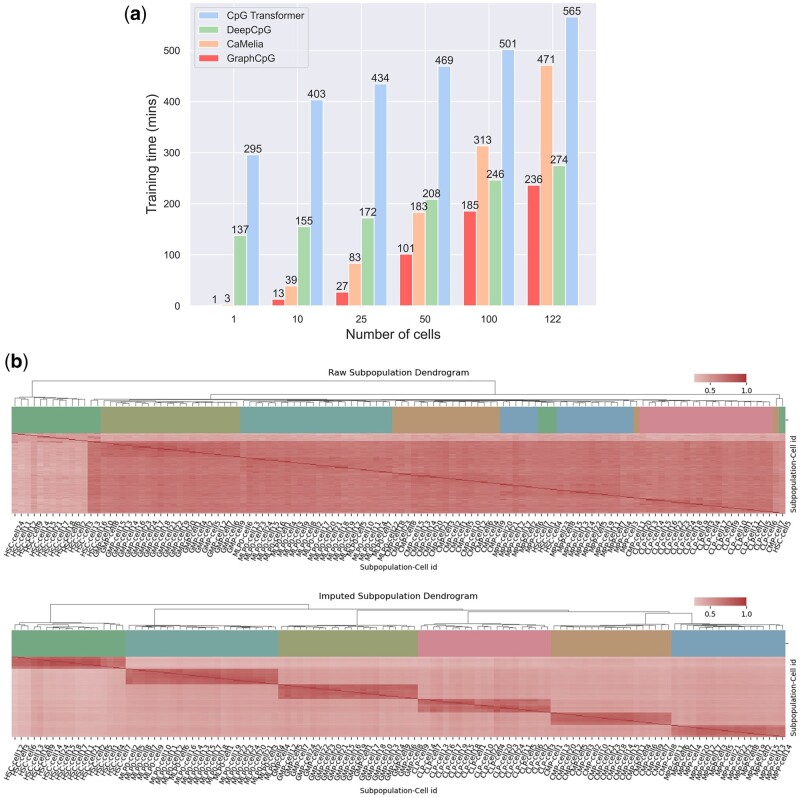
Computation boost and hierarchical clustering analysis. (a) Training time comparison among models with different-size datasets. (b) The hierarchical clustering heatmap and dendrogram of the inter-cellular Spearman similarity matrix based on raw and imputed data from Hemato.

First, we conducted a general training time comparison among all the models with increasing cell numbers. We randomly sampled Hemato and split it into six different-size datasets with 1, 10, 25, 50, 100, and 122 cells, respectively. As shown in Fig. 4a, we measured the time for each model and found that GraphCpG had the shortest time consumption with increasing cell numbers. CaMelia was relatively fast when there were no more than a hundred cells, but its time consumption increased dramatically due to its long preprocessing time. DeepCpG showed a mild increase in time consumption, but its loading huge preprocessing data to random access memory would limit its performance on large datasets. CpG Transformer had the longest time consumption in this comparison, as its model scaled quadratically with the cell number. However, only CpG Transformer and GraphCpG can load datasets at runtime among models here, which are friendly to large datasets because of negligible preprocessing time and low memory consumption.

Second, on recent large datasets such as Neuron-Mouse and Neuron-Homo, GraphCpG and CpG Transformer normally take around one day on training all the loci for one epoch, but we found that their loss in training hardly decreased without using the whole training set of loci in the first epoch. On the largest dataset of this study (Neuron-Homo), with 1% of the training set, both GraphCpG and CpG Transformer achieved results within 0.5% of their best performance (Supplementary Fig. S4). Moreover, with merely 0.01% of the training set, GraphCpG achieved an AUROC of 86.88, which was more efficient than CpG Transformer (61.21). This indicates that we can obtain satisfactory prediction performance by randomly sampling part of the whole sites rather than crawling through the whole epoch. Compared with approximately a hundred times the training time of traversing whole training sites, the rapid convergence of GraphCpG outperforms others in improving performance with limited training time and advances on large datasets.

### 3.4 Downstream analysis

To illustrate GraphCpG’s capacity in cell clustering, cell type identification, and differential methylation analysis, we applied hierarchical clustering analysis and differential methylation analysis related to Hemato.

We conducted the hierarchical clustering analysis, including a heatmap of cell clustering and a dendrogram with cell type. The hierarchical clustering analysis was performed on the both raw and imputed dataset, which contains 21 common lymphoid progenitor (CLP), 19 common myeloid progenitor (CMP), 22 granulocyte macrophage progenitor (GMP), 18 hematopoietic stem cell (HSC), 24 immature lymphoid progenitor 0 (MLP0), and 18 multipotent progenitor (MPP). The inter-cellular Spearman similarity matrix was calculated based on the overlapped loci between each pair of cells. As shown in Fig. 4b, the utilization of imputed data for cell clustering led to more accurate identification of distinct cell types compared to raw data. It also enhanced the visualization and readability of the hierarchical clustering results. Moreover, compared with the raw dataset (Supplementary Fig. S5), the imputed dataset showed a more similar methylation distribution of six subtypes to the reference bulk dataset in differentially methylated regions identified by Farlik *et al.* (2016). In distinguishing myeloid and lymphoid lineages transited from HSC and MPP, the imputed results also follow that DNA methylation levels at regulatory regions are on average lower in myeloid progenitors than in lymphoid progenitors. These indicate that imputation improved the differential methylation analysis of the single-cell dataset (122 single cells) by making it more consistent with the analysis of the bulk dataset (21250-cell sample) and correctly predicting missing methylation levels.

## 4 Discussion

By extracting locus-aware neighboring subgraphs and training optimized graph-based embeddings, with only methylation matrices, GraphCpG obtains state-of-the-art imputation performance over DeepCpG, CaMelia, and CpG Transformer on large datasets having more than hundreds of cell samples such as Hemato, Neuron-Mouse, and Neuron-Homo. Though dropping absolute methylation positions and DNA context in training, GraphCpG still obtains competitive performance on smaller datasets such as HCC and MBL. Combining the bipartite graph, the visualization of optimized embeddings of cell nodes and loci nodes enables analyses for inter-cellular and inter-loci similarity on interested regions. With lower cost on training time, GraphCpG demonstrates scalability on larger datasets with more than hundreds of cell samples and potentiality in downstream analysis.

The proposed neighboring subgraph extraction and locus-aware encoding enlighten a new representation, which identifies sequential neighboring locus to describe the target methylation state within an abstract target cell. However, the small window size would limit the scope and hinder the performance of the method. The expansion of neighboring subgraph window size is a topic worth analysis. Further improvement in graph sampling or neighborhood aggregation would also enlarge the view of the neighboring subgraph, which could be future work.

Theoretically, GraphCpG is able to accurately impute any new single-cell methylome data. It scales well with the number of cells increasing. Researchers could impute interested sites quickly with retrievable visualization. In addition, it also aids them in saving training time and conducting downstream analysis.

## Supplementary Material

btad533_Supplementary_Data
