# Supplementary material for: GraphCpG: imputation of single-cell methylomes based on locus-aware neighboring subgraphs
Source: Bioinformatics. 2023 Aug 30;39(9):btad533. doi: 10.1093/bioinformatics/btad533 (PMC10516632; doi:10.1093/bioinformatics/btad533)

Supplementary information for GraphCpG: Imputation of Single-cell Methylomes Based on Locus-aware Neighboring Subgraphs

Yuzhong Deng^1^, Jianxiong Tang^1^, Jiyang Zhang^1^, Jianxiao Zou^3,1^, Que Zhu^2,^* and Shicai Fan^3,1,^*

^1^School of Automation Engineering, University of Electronic Science and Technology of China, Chengdu, 611731, Sichuan, China, ^2^Department of Out-patient, The Second Affiliated Hospital of Chongqing Medical University, 400010, Chongqing, China, ^3^Shenzhen Institute for Advanced Study, University of Electronic Science and Technology of China, Shenzhen, 518110, Guangdong, China.

**Table 1. Summary of dataset statistics.**

| Datasets | cells | Sum CpG Sites | Observed Sites | Sparsity^a^ (%) | Methylation Rate^b^(%) | Genome Build | |
| --- | --- | --- | --- | --- | --- | --- | --- |
| HCC | 25 | 2044635 | 5891193 | 88.47 | 29.80 | hg19 | |
| MBL | 30 | 4779569 | 13155172 | 90.82 | 52.79 | hg19 | |
| Hemato | 122 | 18050756 | 34855325 | 98.41 | 79.67 | hg38 | |
| Neuron-Mouse | 690 | 19974995 | 414017690 | 96.99 | 75.47 | mm10 | |
| Neuron-Homo | 780 | 26977898 | 746556608 | 96.45 | 79.45 | hg38 | |
| Datasets | **chr1** | **chr2** | **chr3** | **chr4** | **chr5** | **chr6** | |
| HCC | 202119 | 134413 | 93557 | 68817 | 85750 | 98968 | |
| MBL | 421908 | 335863 | 239822 | 214611 | 227915 | 224610 | |
| Hemato | 1516890 | 1368528 | 1017897 | 893829 | 933570 | 944940 | |
| Neuron-Mouse | 1385803 | 1435740 | 1110719 | 1237493 | 1273738 | 1091716 | |
| Neuron-Homo | 2187593 | 2089730 | 1589335 | 1429005 | 1452153 | 1441214 | |
| Datasets | **chr7** | **chr8** | **chr9** | **chr10** | **chr11** | **chr12** | |
| HCC | 136199 | 81302 | 105101 | 88353 | 102077 | 82429 | |
| MBL | 281674 | 200004 | 235018 | 221318 | 231338 | 222109 | |
| Hemato | 1016593 | 835550 | 766449 | 890862 | 864507 | 841386 | |
| Neuron-Mouse | 1114832 | 1070910 | 1021433 | 1044574 | 1122011 | 887233 | |
| Neuron-Homo | 1493012 | 1256289 | 1122331 | 1300191 | 1260757 | 1249779 | |
| Datasets | **chr13** | **chr14** | **chr15** | **chr16** | **chr17** | **chr18** | |
| HCC | 40831 | 64958 | 56914 | 87358 | 118294 | 41029 | |
| MBL | 113450 | 150252 | 155754 | 243028 | 284082 | 101804 | |
| Hemato | 518798 | 557371 | 558313 | 746429 | 829719 | 450163 | |
| Neuron-Mouse | 918771 | 848435 | 827707 | 708091 | 805292 | 683930 | |
| Neuron-Homo | 785240 | 837467 | 816170 | 1035079 | 1115902 | 663392 | |
| Datasets | **chr19** | **chr20** | **chr21** | **chr22** | **chrX** | **chrY** | **chrM** |
| HCC | 146476 | 67558 | 31912 | 58840 | 48300 | 3080  NaN  43211  77842  102342 | NaN  NaN  435  NaN  NaN |
| MBL | 322368 | 139530 | 69907 | 143204 | NaN |  |  |
| Hemato | 770319 | 517941 | 245978 | 411800 | 509278 |  |  |
| Neuron-Mouse | 537466 | NaN | NaN | NaN | 771259 |  |  |
| Neuron-Homo | 1030170 | 705709 | 367793 | 548299 | 1098946 |  |  |

^a^ The sparsity is the proportion of unobserved sites on all the sites.

^b^ The methylation rate is calculated by methylated sites over observed sites.

**Table 2. Width of the sliding window for GraphCpG.**

|  | Window Size | | | | | | \| **Variation** \| \| --- \| |
| --- | --- | --- | --- | --- | --- | --- | --- | --- |
|  | **11** | **21** | **25** | **31** | **41** | **51** |  |
| HCC | 96.94 | 96.94 | **96.99** | 96.9 | 96.95 | 96.94 | 0.00052 |
| MBL | 89.55 | **89.73** | 89.66 | 89.54 | 89.64 | 89.57 | 0.00555 |
| Hemato | 89.61 | 89.69 | 89.76 | 89.75 | **89.77** | 89.75 | 0.00378 |

**Table 3. Training parameters**

**DeepCpG** We：

- Enlarged batch size to 512 on all the datasets

**CpG Transformer** We：

- Used more chromosomes in holdout validation and testing.
- Reduced the window size to avoid running out of GPU memory on Neuron-Mouse and Neuron-Homo.

| Datasets | Cells | Batch Size | | Window Size | |
| --- | --- | --- | --- | --- | --- |
|  |  | **CpG Transformer** | **GraphCpG** | **CpG Transformer** | **GraphCpG** |
| HCC | 25 | 1 | 5120 | 1024 | 25 |
| MBL | 30 | 1 | 10240 | 1024 | 21 |
| Hemato | 122 | 1 | 5120 | 1024 | 41 |
| Neuron-Mouse | 690 | 1 | 512 | 21 | 21 |
| Neuron-Homo | 780 | 1 | 512 | 21 | 21 |

**Table 4. The performance comparison of GraphCpG with other methods on different datasets.**

| Dataset | Cell Num | macroF1 | | | |  | Balanced accuracy | | | |
| --- | --- | --- | --- | --- | --- | --- | --- | --- | --- | --- |
|  |  |  | Deep Learning | | |  |  | Deep Learning | | |
|  |  | CaMelia | DeepCpG | CpG  Transformer | GraphCpG |  | CaMelia | DeepCpG | CpG  Transformer | GraphCpG |
| HCC | 25 | 87.89 | 84.4 | **88.88** | 86.54 |  | 92.12 | 89.2 | **92.49** | 92.03 |
| MBL | 30 | 84.27 | 81.84 | **86.88** | 84.47 |  | 81.48 | 79.96 | **85.17** | 82.25 |
| Hemato | 122 | 94.32 | 94.43 | 94.33 | **94.61** |  | 78.92 | 78.41 | 78.77 | **79.1** |
| Neuron-Mouse | 690 | 93.28 | 87.58 | 93.42 | **93.48** |  | 81.39 | 80.9 | 81.26 | **81.63** |
| Neuron-Homo | 780 | 95.53 | 95.18 | 95.59 | **95.62** |  | 84.06 | 83.28 | 84.11 | **84.17** |

*Note*: Bold numbers indicate the best performance.

**Fig. 1 Boxplots of AUROC per cell on all the datasets.**


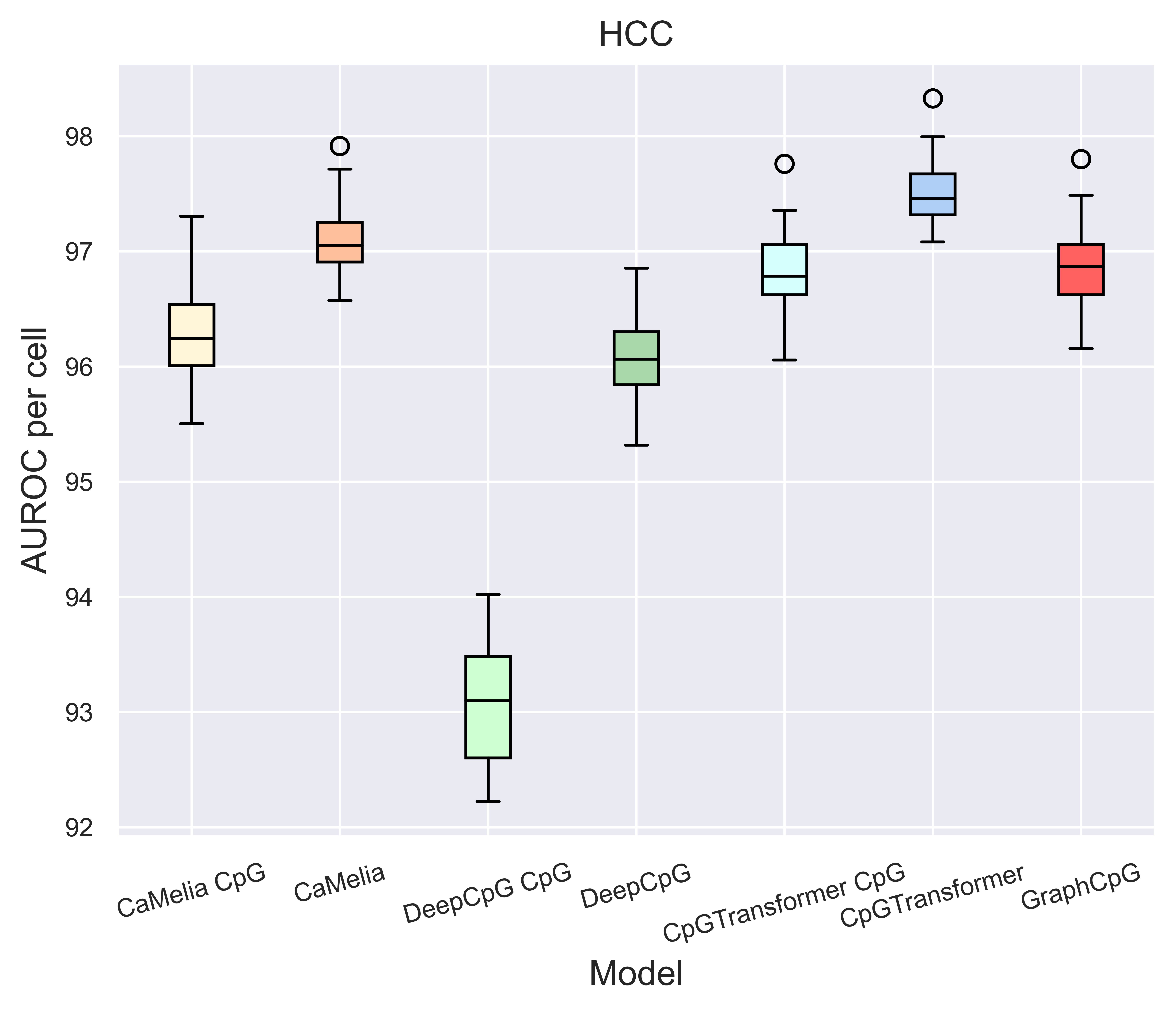

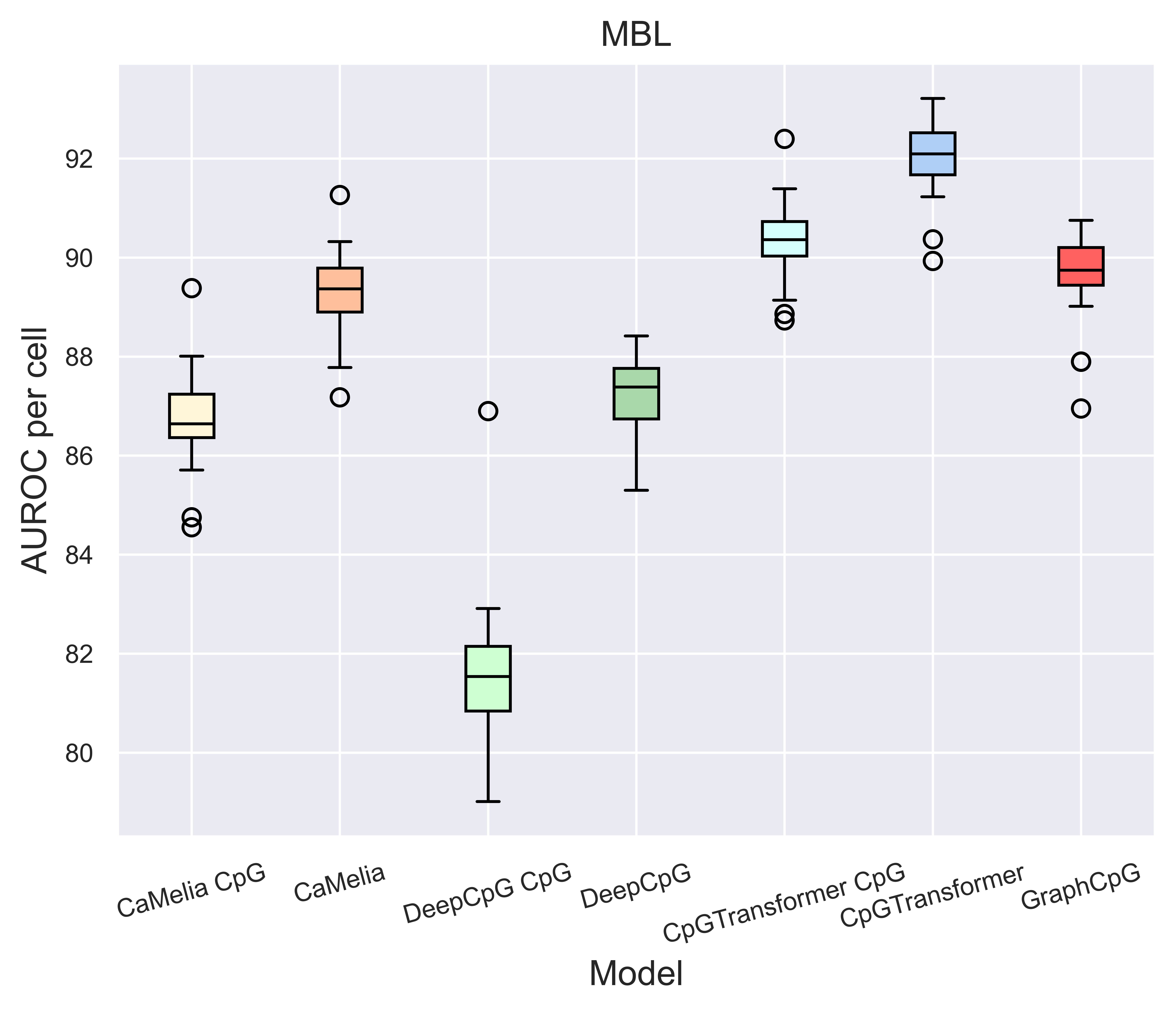

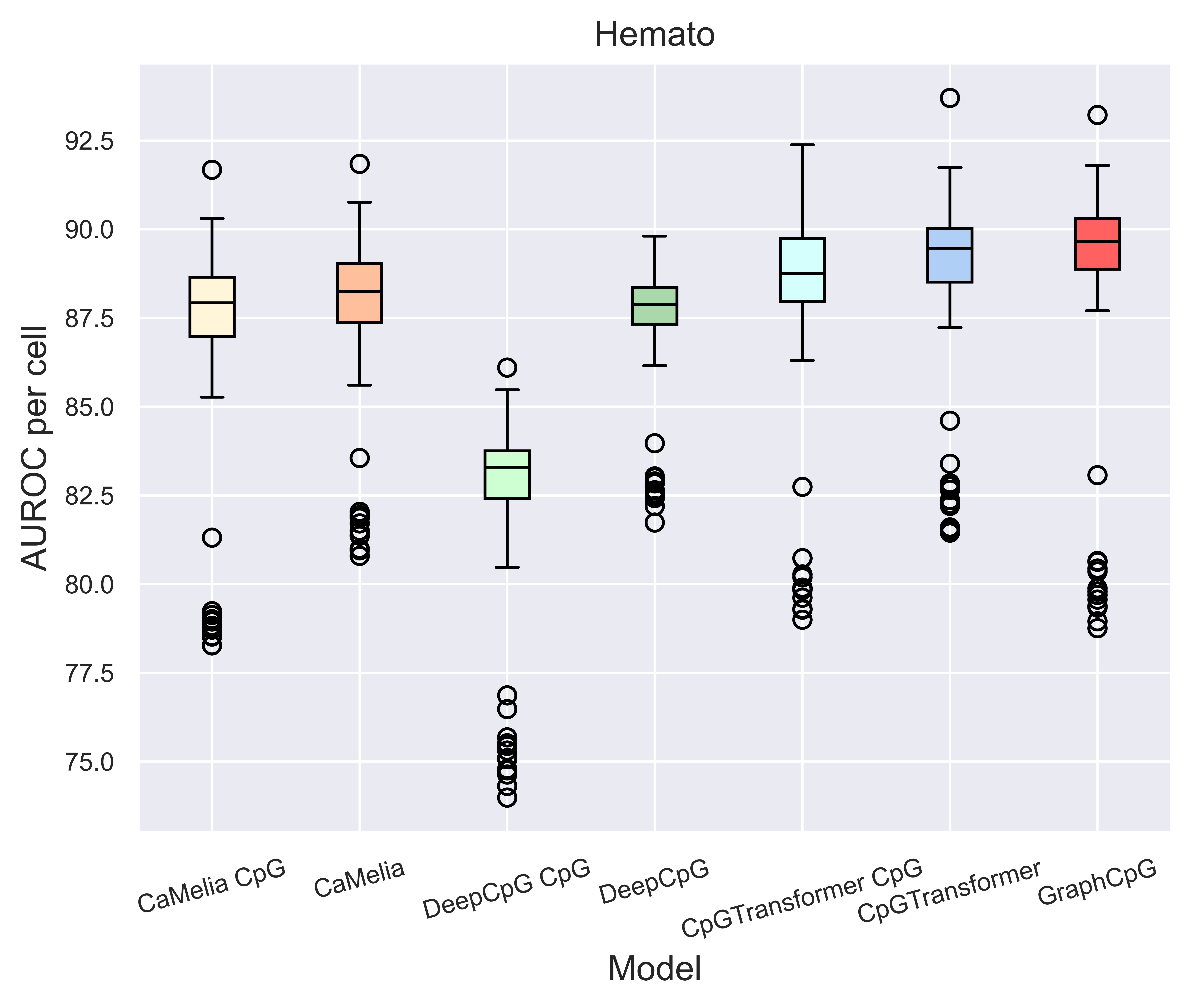


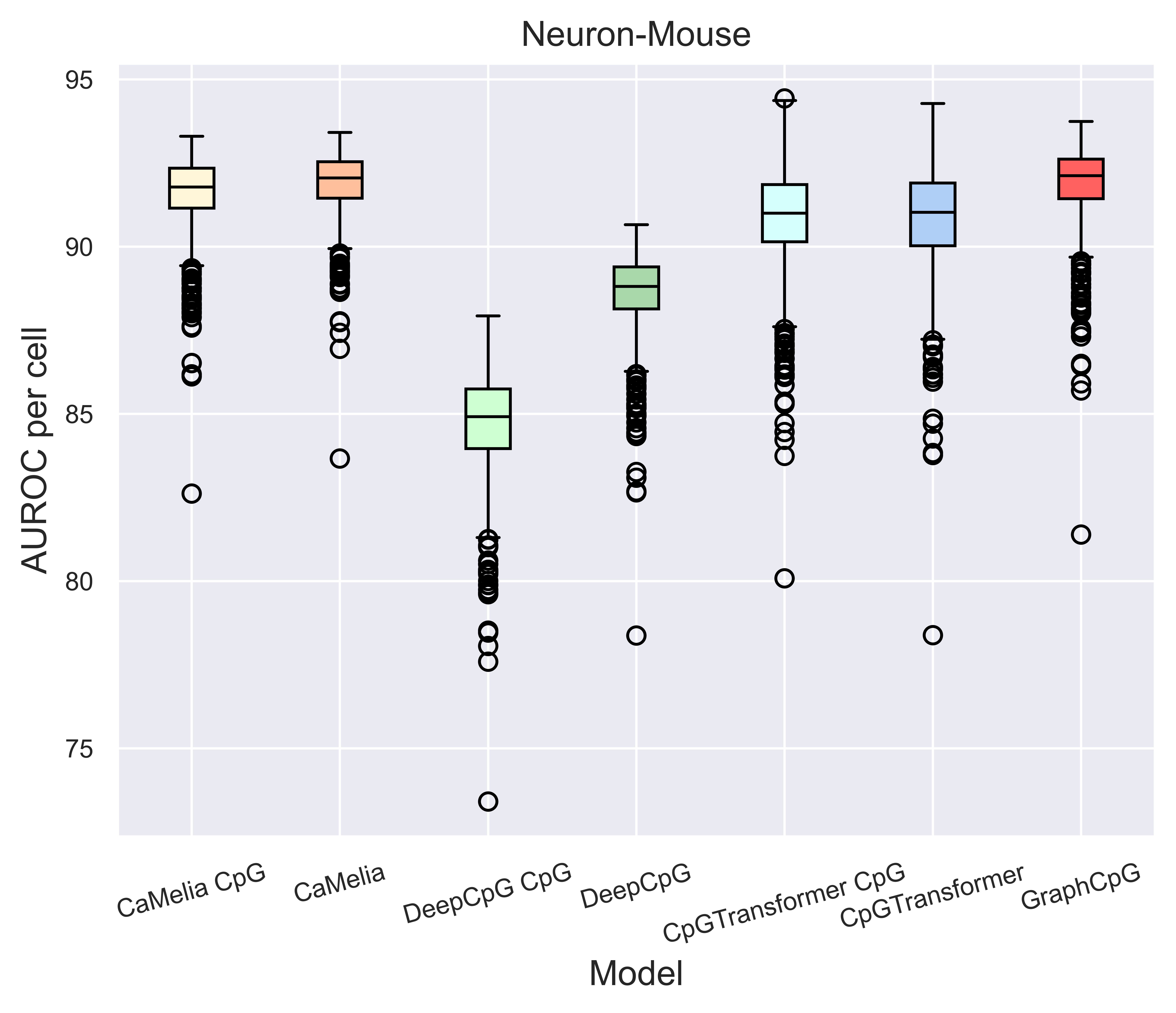

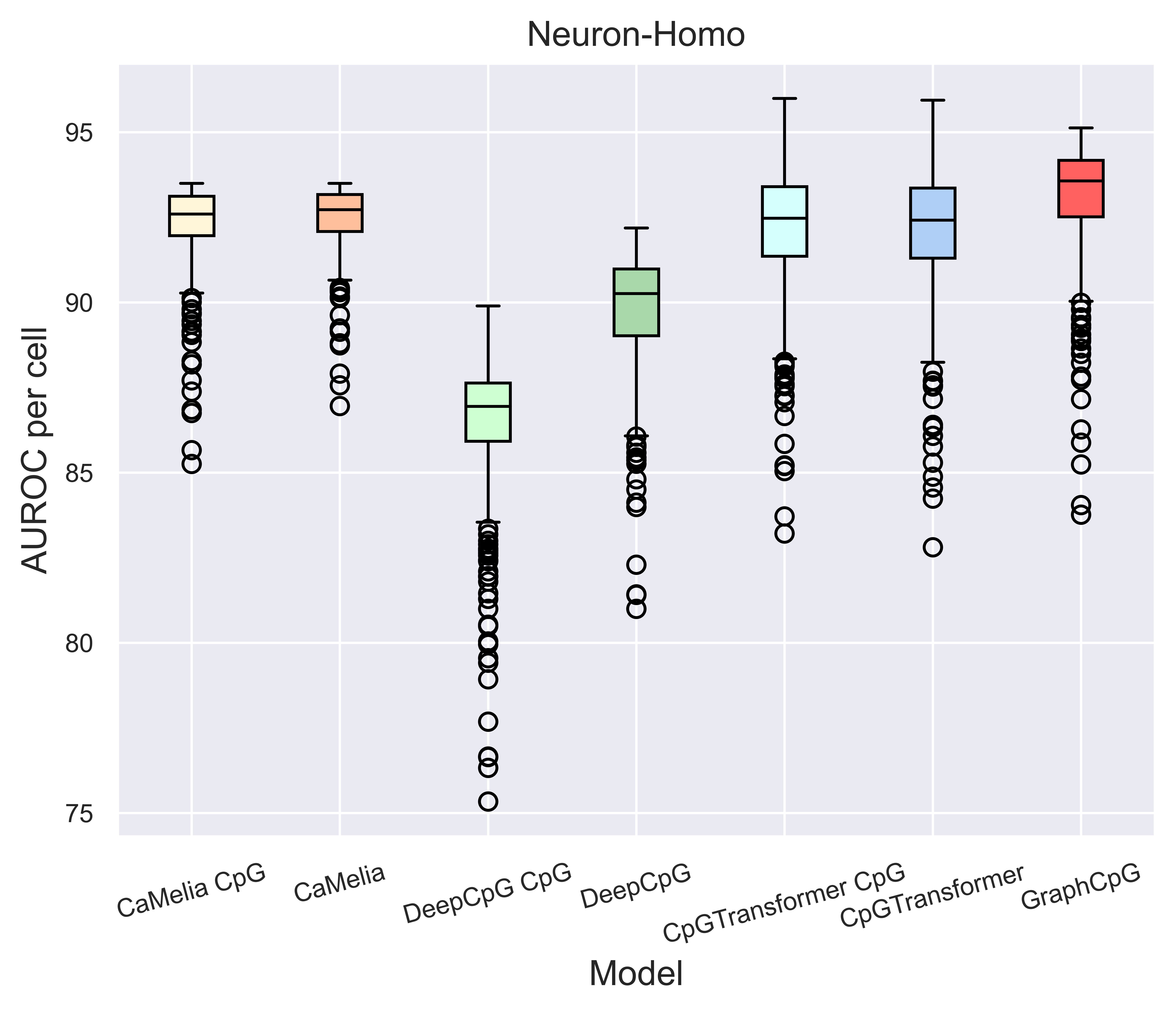


**Fig. 2 Boxplots of AUROC per cell on Hemato dataset.** Groups are stratified by genomic contexts. Genomics contexts are based on GRCh38.


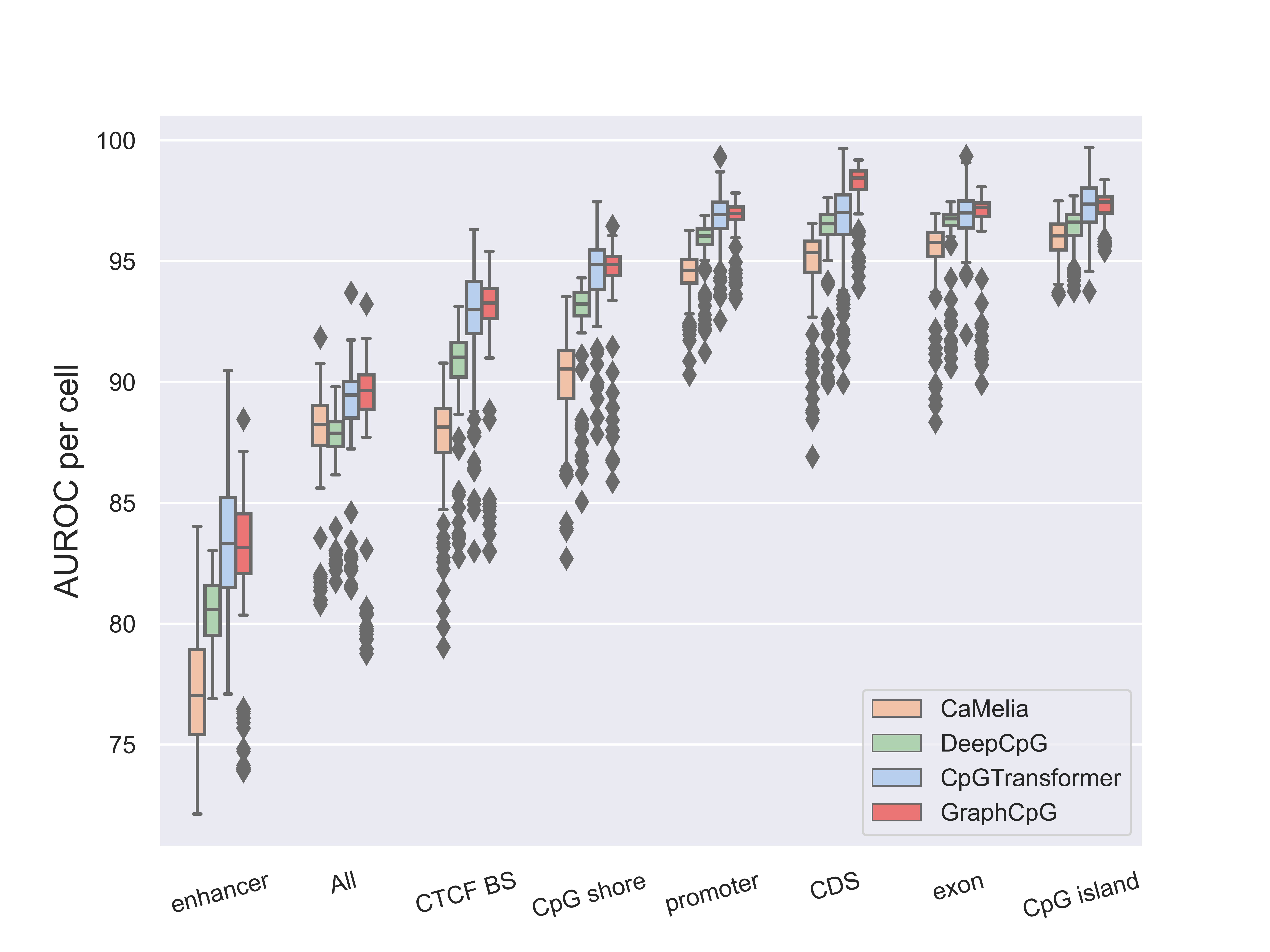


**Fig. 3 Visualization of neighboring subgraphs from Hemato.** For bipartite graphs in each row, the left sides are cells dyed in white, and the right sides are sequential loci dyed in a blue gradient. The upper graph represents a methylated target site and the lower graph profiles an unmethylated target site.


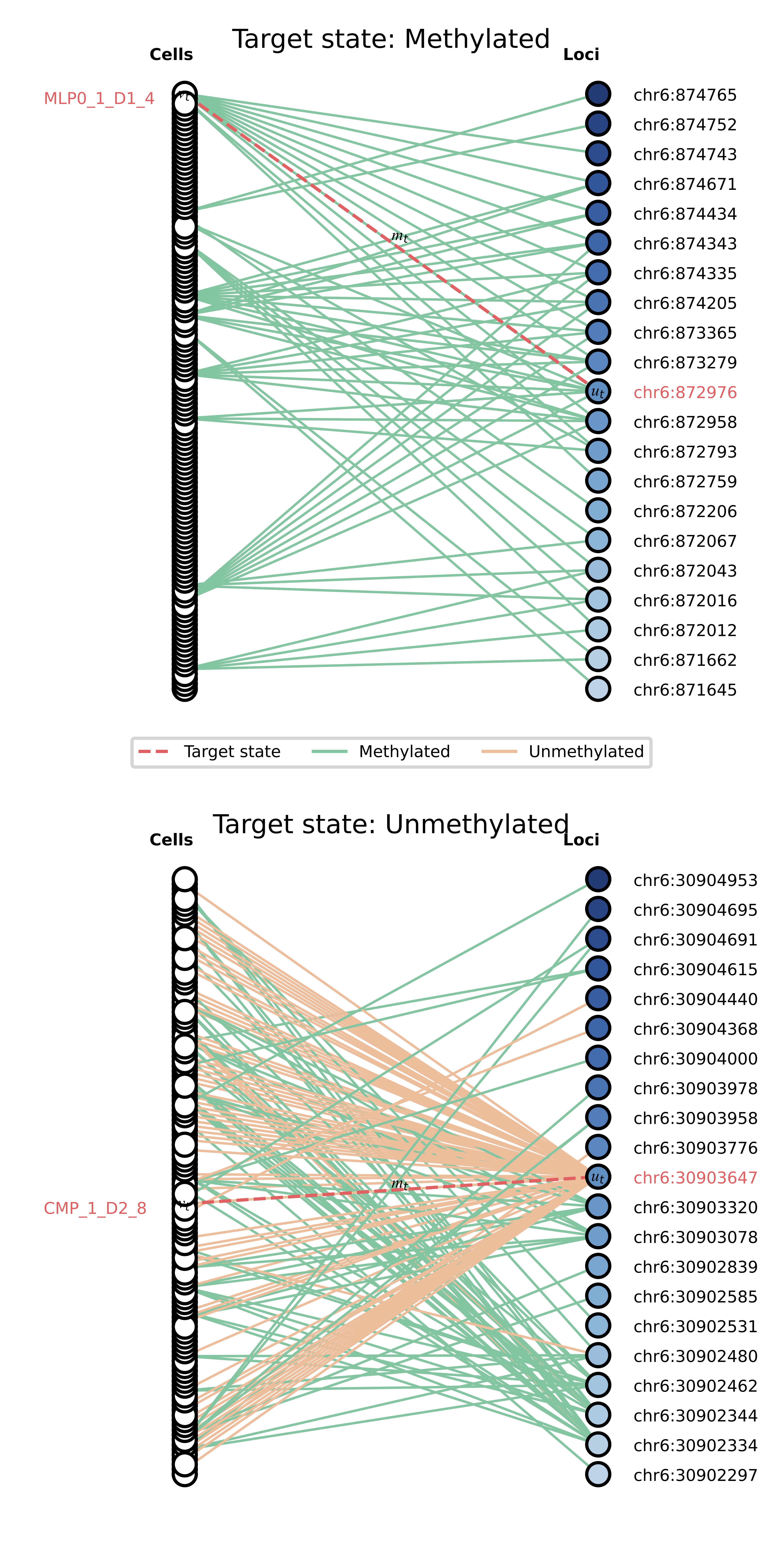


**Fig. 4 Comparison of performance between GraphCpG and CpG Transformer on the test set with increasing proportion of training set within the first epoch.**


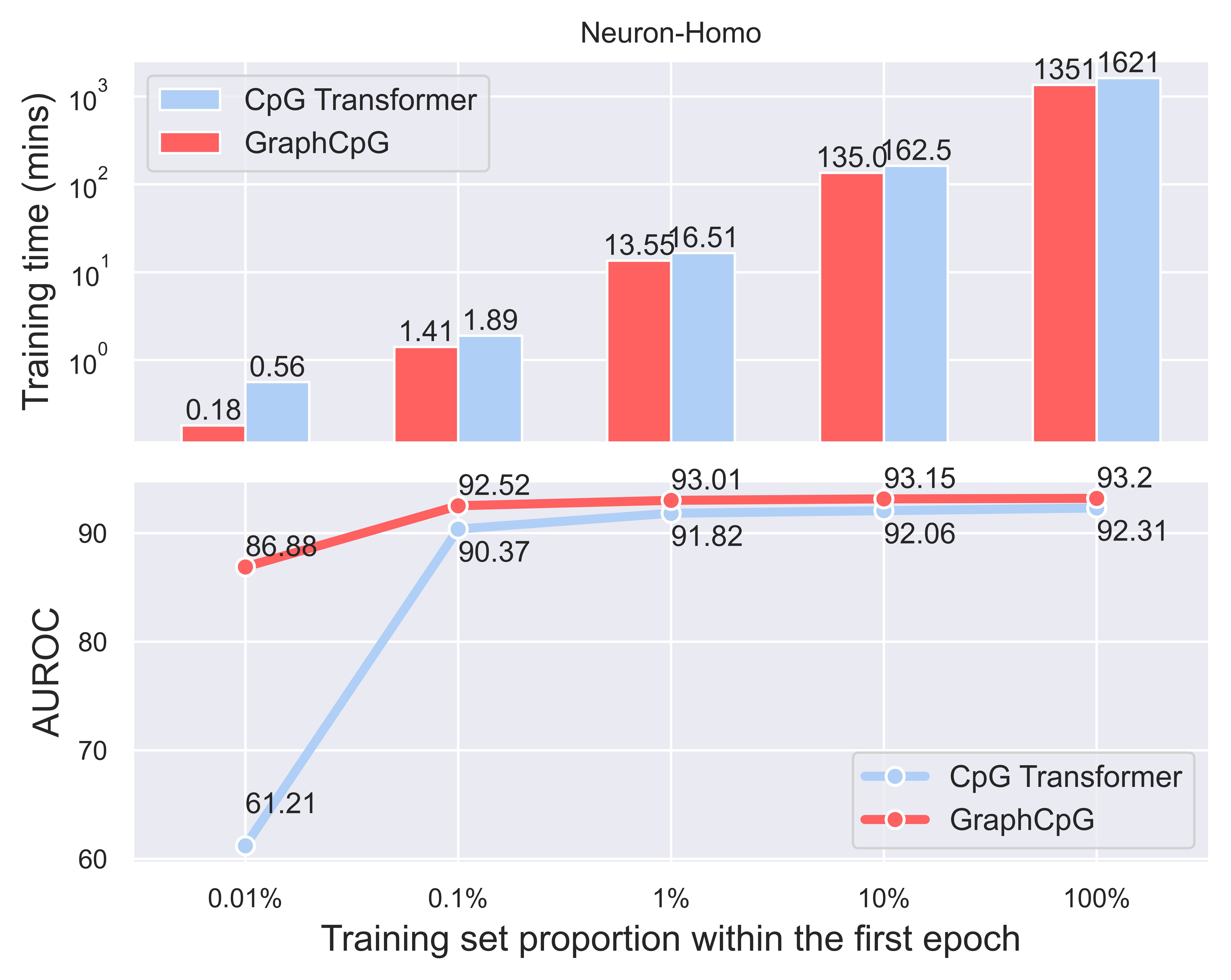


**Fig. 5 Boxplots and swarm plots of average methylation level by cells in 1234 regulatory regions on raw, imputed, and bulk datasets.** ***p ≤ 0.001 (two-tailed Wilcoxon test). Genomics contexts are based on GRCh38.


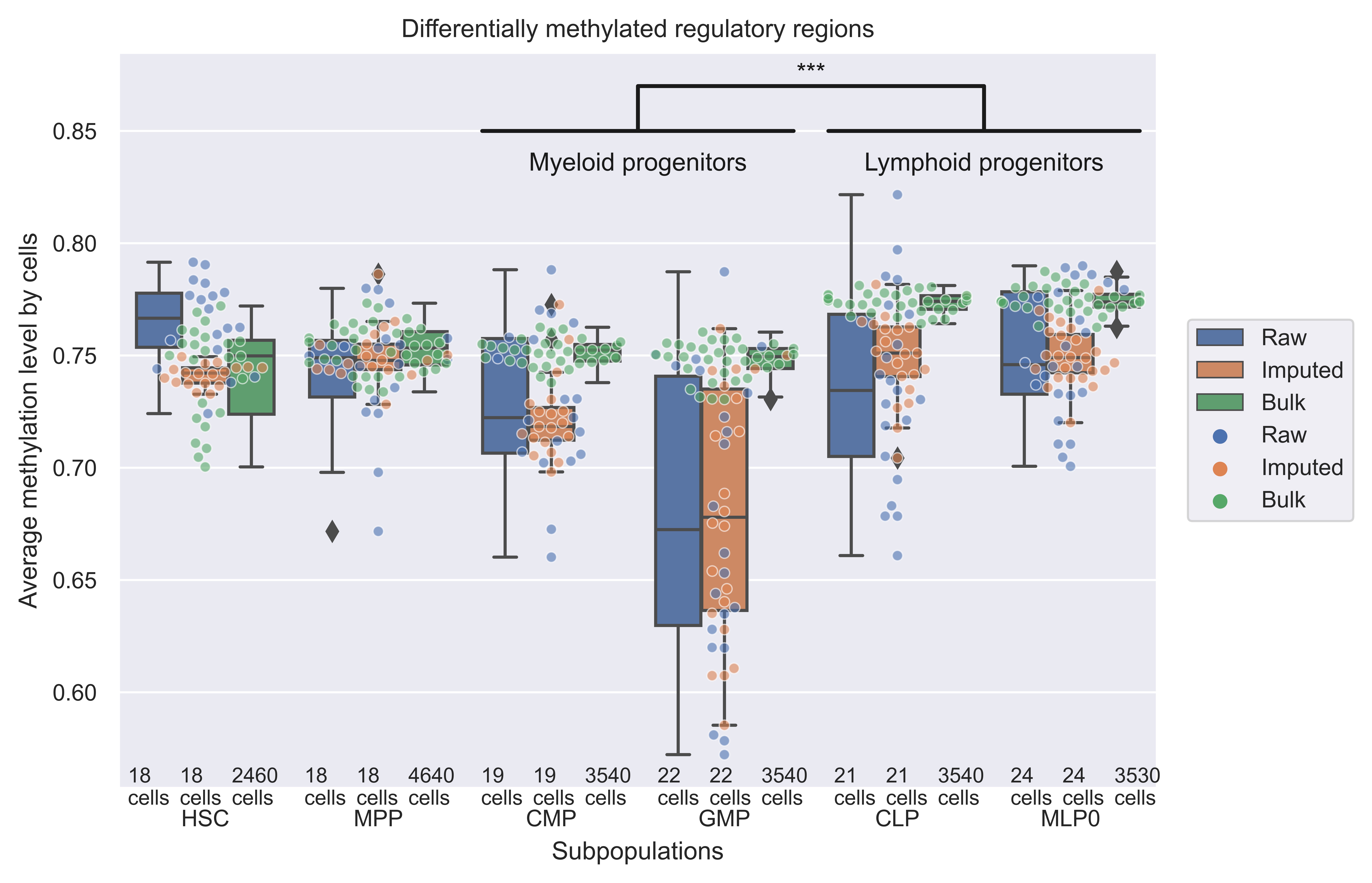

Supplement: btad533_Supplementary_Data [file btad533_supplementary_data.zip › suppl230826Clean.docx]
