# Supplementary figures and images for: GraphCpG: imputation of single-cell methylomes based on locus-aware neighboring subgraphs
Source: Bioinformatics. 2023 Aug 30;39(9):btad533. doi: 10.1093/bioinformatics/btad533 (PMC10516632; doi:10.1093/bioinformatics/btad533)

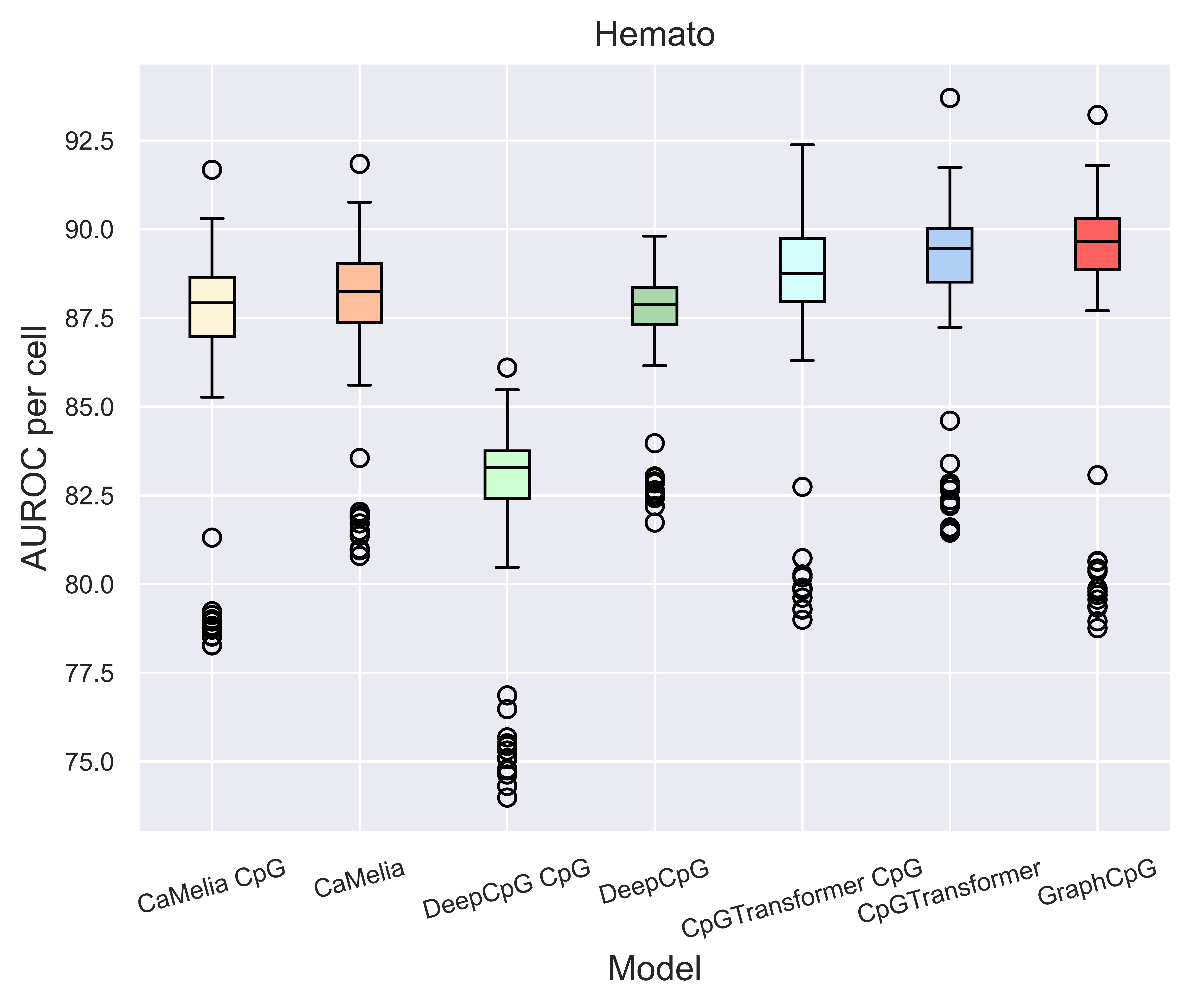

Supplement: btad533_Supplementary_Data [file btad533_supplementary_data.zip › suppl_Figure_1_Hemato.jpg]

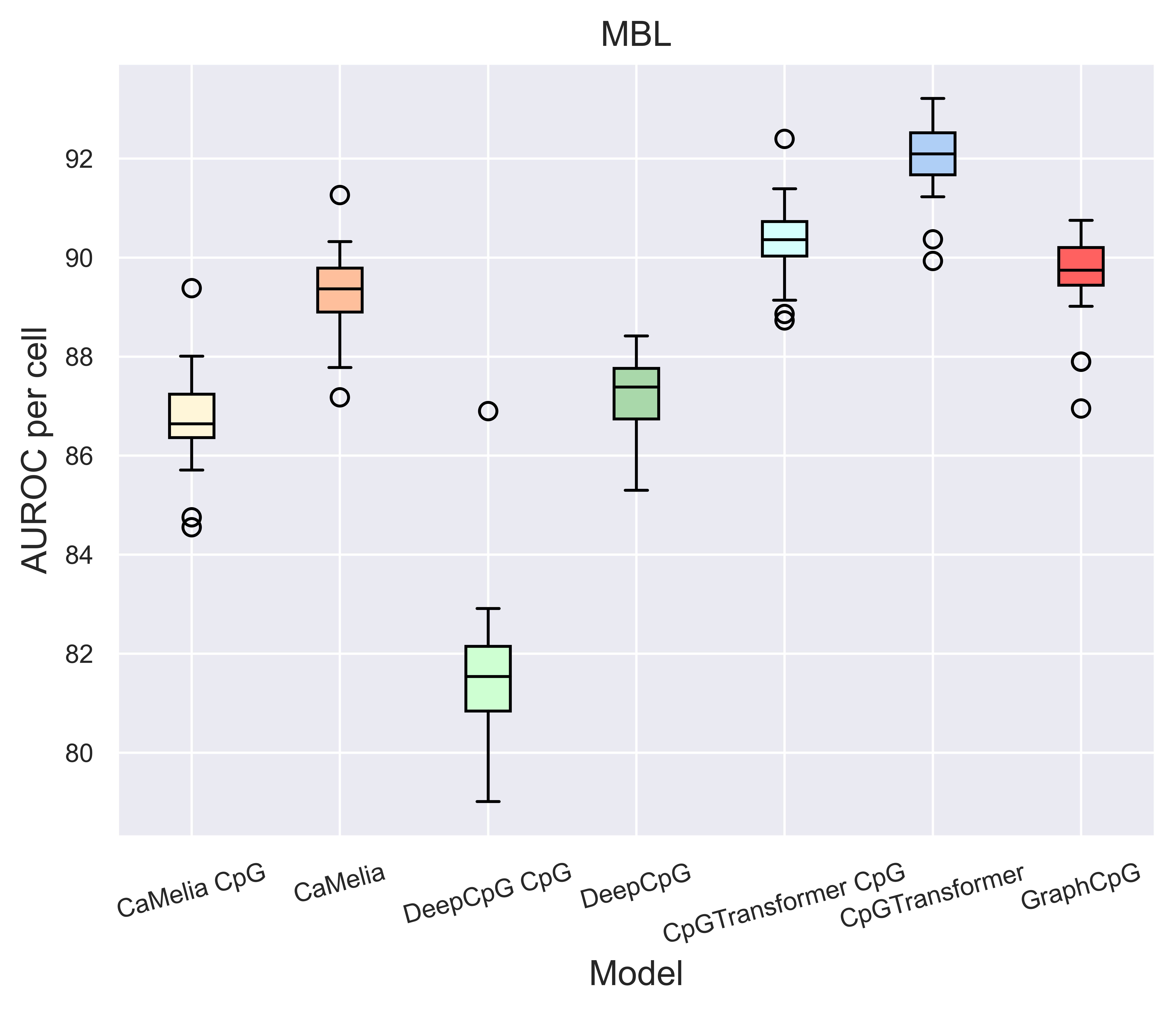

Supplement: btad533_Supplementary_Data [file btad533_supplementary_data.zip › suppl_Figure_1_MBL.jpg]

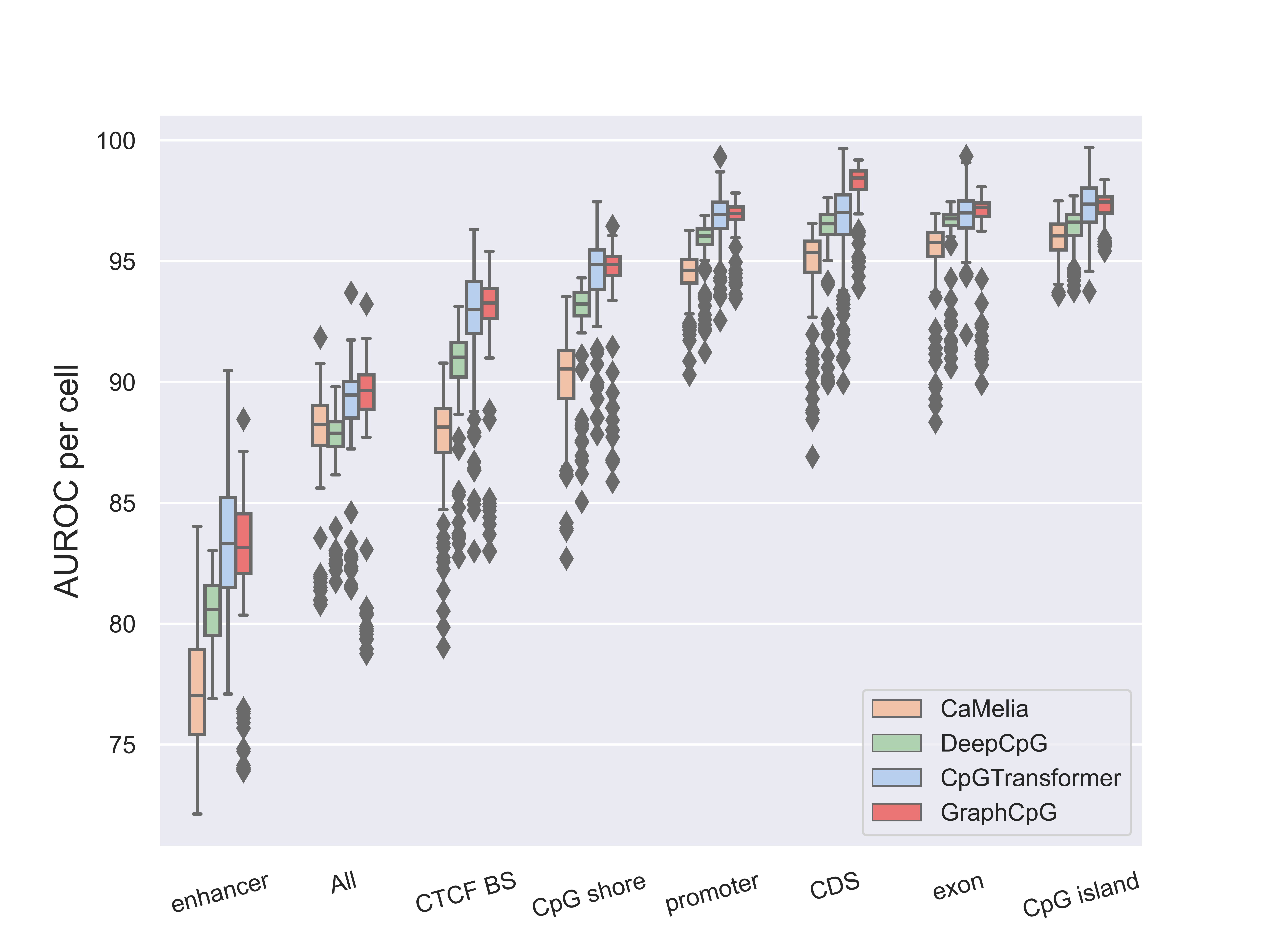

Supplement: btad533_Supplementary_Data [file btad533_supplementary_data.zip › suppl_Figure_2.jpg]

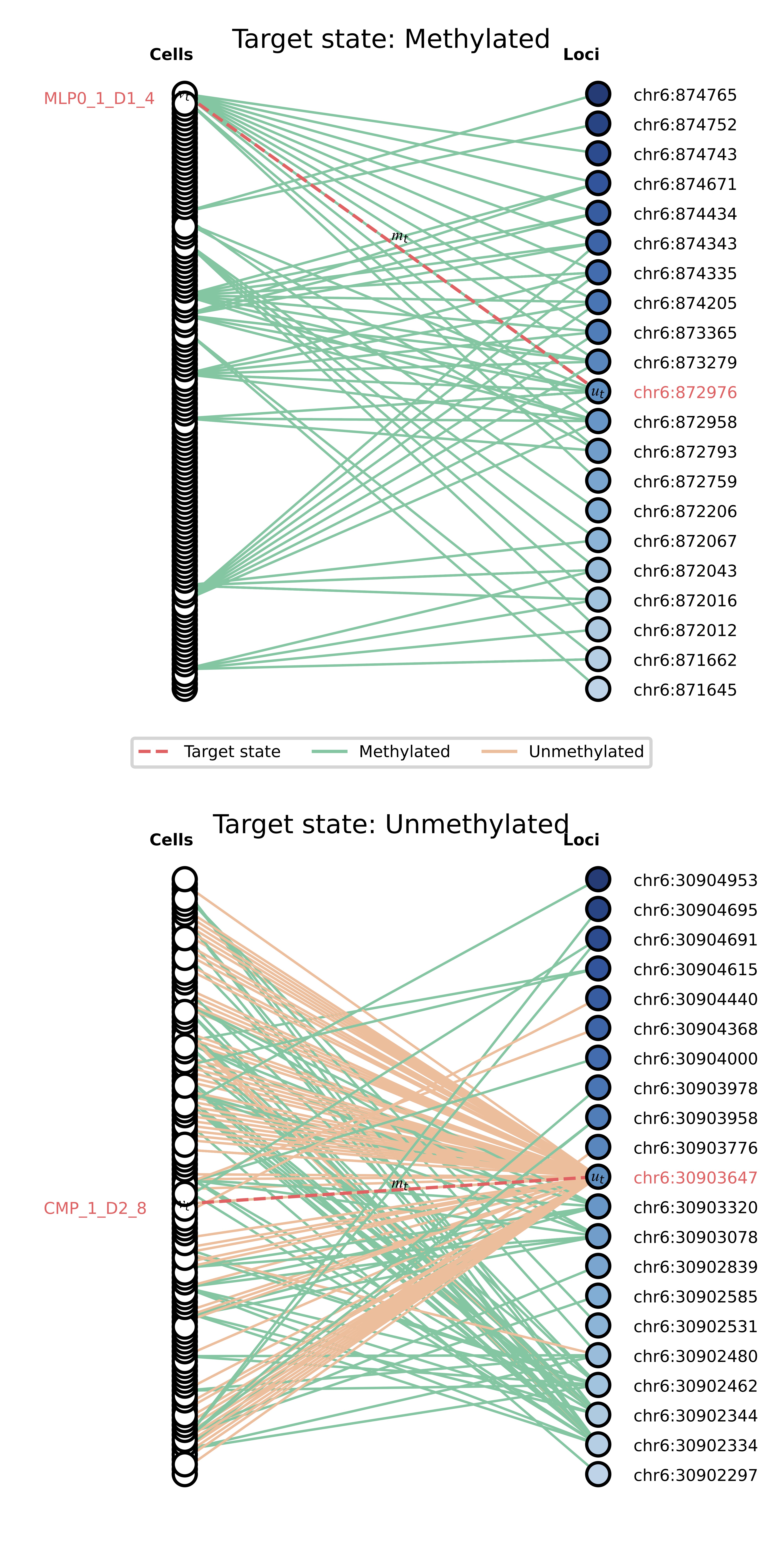

Supplement: btad533_Supplementary_Data [file btad533_supplementary_data.zip › suppl_Figure_3.jpg]

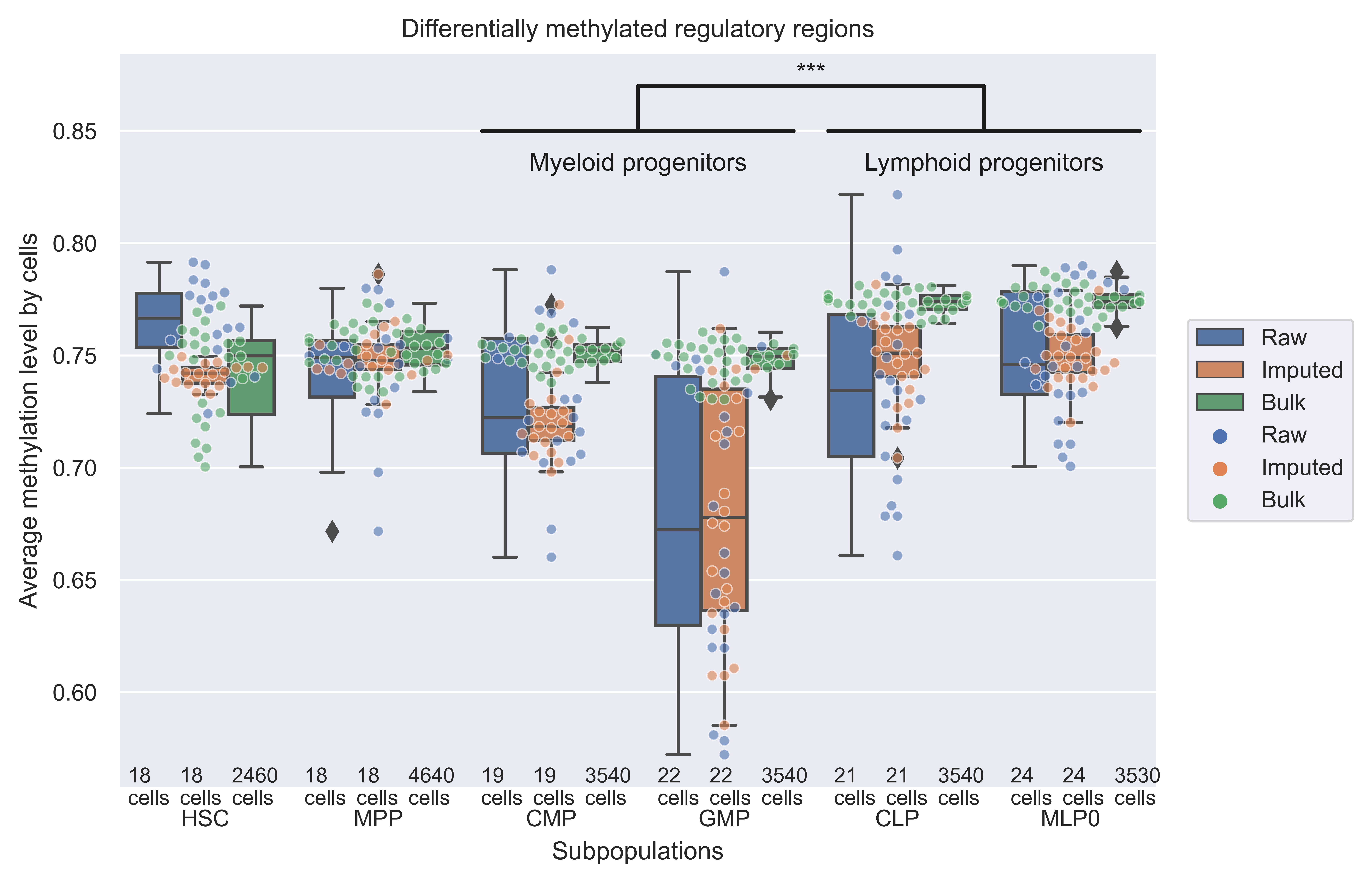

Supplement: btad533_Supplementary_Data [file btad533_supplementary_data.zip › suppl_Figure_5.jpg]

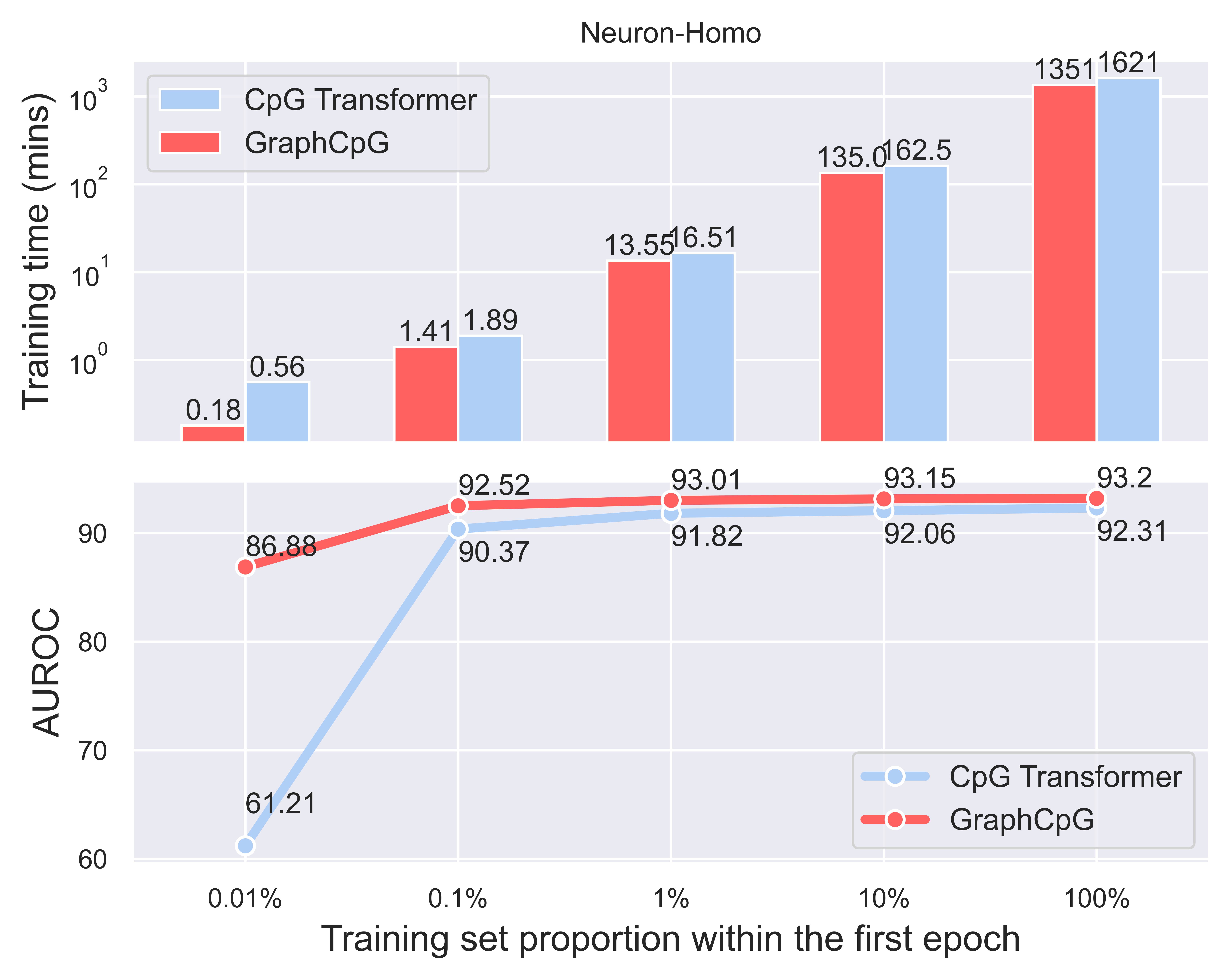

Supplement: btad533_Supplementary_Data [file btad533_supplementary_data.zip › suppl_Figure_4.jpg]

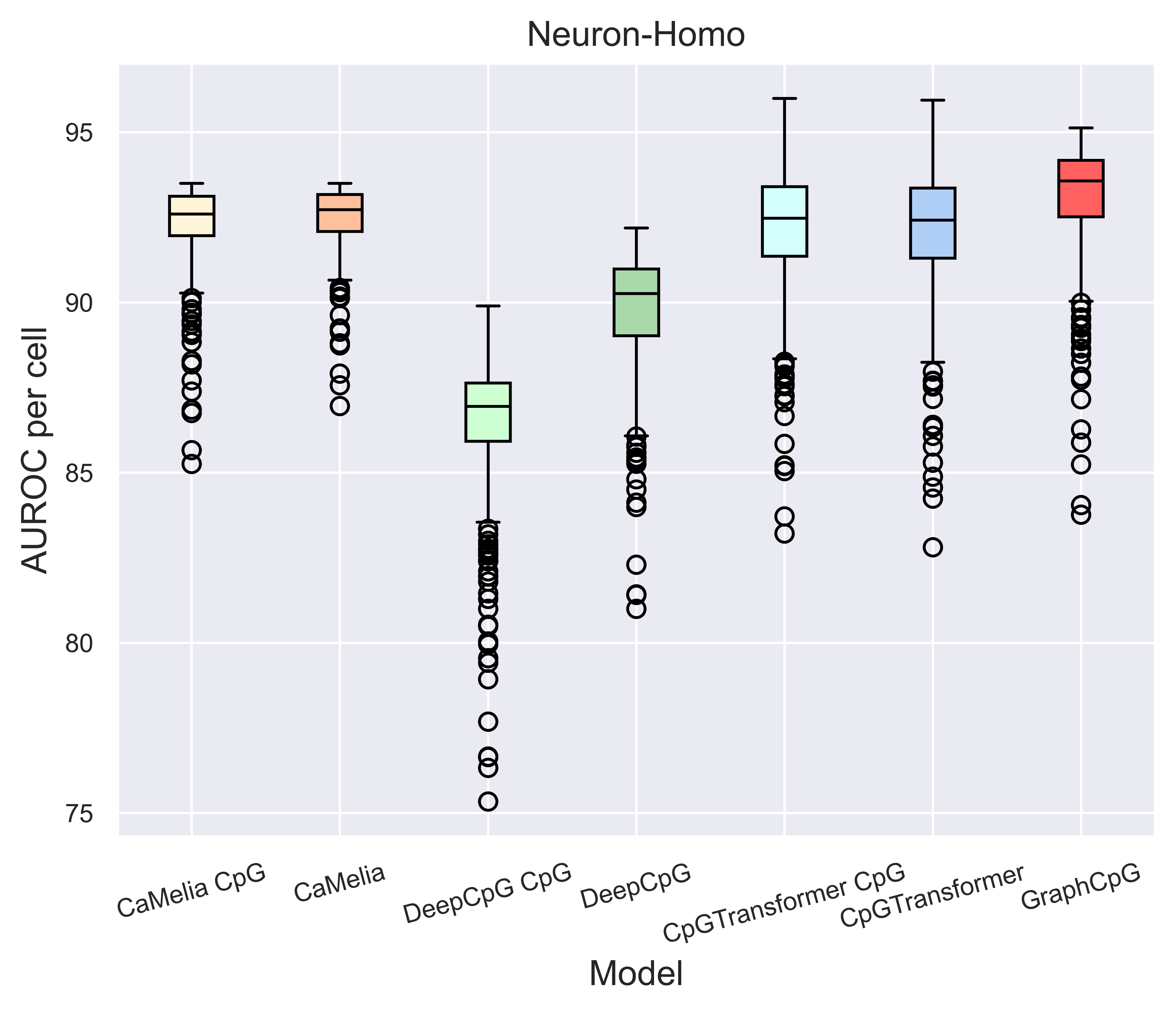

Supplement: btad533_Supplementary_Data [file btad533_supplementary_data.zip › suppl_Figure_1_Neuron_Homo.jpg]

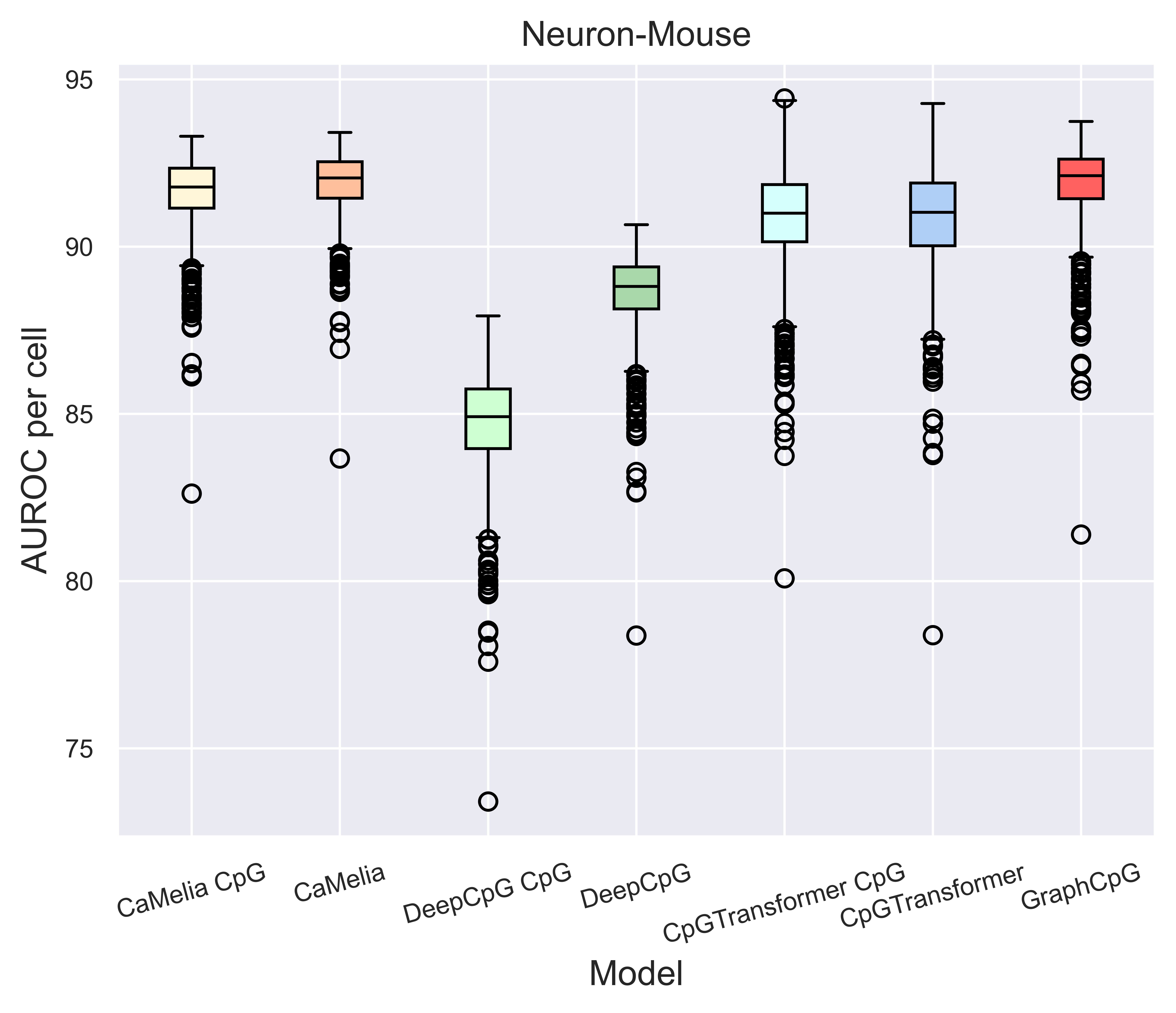

Supplement: btad533_Supplementary_Data [file btad533_supplementary_data.zip › suppl_Figure_1_Neuron_Mouse.jpg]
